# The chitinolytic enzymes from the entomopathogenic fungus *Metarhizium anisopliae*: GH family distribution, domain architecture, and protein regulation

**DOI:** 10.3389/ffunb.2026.1732437

**Published:** 2026-02-17

**Authors:** Qusai Al Abdallah, Kholoud M. Alananbeh, Nehaya Al-Karablieh, Salah-Eddin Araj

**Affiliations:** 1 Department of Agricultural Biotechnology and Genetic Engineering, Faculty of Agricultural Technology, Al-Ahliyya Amman University, Amman, Jordan; 2 Department of Plant Protection, School of Agriculture, The University of Jordan, Amman, Jordan; 3 Hamdi Mango Center for Scientific Research, The University of Jordan, Amman, Jordan

**Keywords:** chitin, chitinases, entomopathogenicity, GH families, *Metarhizium anisopliae*, regulatory mechanisms, β-N-acetylglucosaminidases

## Abstract

*Metarhizium anisopliae* is an entomopathogenic fungus that is widely used in the biological control of agricultural pests. During host infection, *M. anisopliae* secretes an arsenal of hydrolytic enzymes such as proteases and chitinases that allow for cuticle penetration and host colonization. The degradation of chitin into N-acetylglucosamine (GlcNAc) monomers is carried out by chitinases and β-N-acetylglucosaminidases (NAGases). Chitin-degrading enzymes of *M. anisopliae* have been the subject of extensive research; however, these studies have not been consolidated into a comprehensive review. This review highlights our current knowledge of the chitinolytic enzymes from *M. anisopliae*, emphasizing the classification of these enzymes based on their mode of action and domain architecture. *M. anisopliae* possesses a set of 21 chitinases that are classified into four glycoside hydrolase family 18 (GH18) subgroups: A, B, C, and D. In addition, *M. anisopliae* produces two GH20 and two GH3 NAGases. The domain architectures of chitinases and NAGases from *M. anisopliae* are highly similar to those found in *Trichoderma* spp. but exhibit some evolutionary distinctions. Moreover, this review integrates insights from other entomopathogenic fungi to identify molecular mechanisms underlying the expression of chitinase and NAGase genes in *M. anisopliae*. The regulatory mechanisms underlying gene expression of chitinolytic enzymes are complex and involve several regulators and metabolic pathways. Finally, chitinases have the potential to be used as biopesticides against fungal pathogens and pest infestations.

## 1 Introduction

Entomopathogenic fungi play an important role in the biological control of agricultural pests. They can cause diseases in insect populations and thereby improve crop yields. In addition, these fungi inhabit a wide range of habitats, enabling effective control of various pest species under field conditions (Moazami, 2011; Kumar et al., 2019).

Entomopathogenic fungi infect their insect hosts through a process that is broadly comparable to that used by plant-pathogenic fungi (reviewed by Ma et al., 2024). Infection begins when conidia attach to the insect cuticle and germinate to produce a germ tube. The germ tube tip then differentiates to form a specialized infection structure, designated appressorium, which secretes cuticle-degrading enzymes, including chitinases, proteases, and lipases, allowing for fungal penetration into the hemocoel (Khachatourians and Qazi, 2008; Santi et al., 2010a; Chethana et al., 2021; Gebremariam et al., 2022). Following successful invasion, the fungus proliferates within the host, producing mycotoxins and hydrolytic enzymes while assimilating host-derived nutrients, ultimately leading to insect death (Butt et al., 2016). Although several thousand entomopathogenic fungal species are thought to exist, only a small proportion has been studied in detail (Blackwell, 2011; Santos et al., 2022). Among these, *Beauveria bassiana* and *Metarhizium anisopliae* remain the most extensively investigated species (Santos et al., 2022).

*M. anisopliae* is a saprophytic fungus that thrives on decaying organic matter and inhabits the soil of a wide range of habitats (Moonjely and Bidochka, 2019; Tang et al., 2025). The taxonomy of *M. anisopliae* has been recently addressed by few articles (Bischoff et al., 2009; Kobmoo et al., 2024; Nishi, 2024). In addition, it is a generalist entomopathogenic fungus that infects a broad spectrum of host insects (Bidochka et al., 1998; Schrank and Vainstein, 2010; St. Leger and Wang, 2020; de Miranda et al., 2024). During different stages of the disease cycle, *M. anisopliae* produces specialized cells such as appressorium, blastospores, and hyphae, which are used for host infection. The disease cycle of *M. anisopliae* is illustrated in Figure 2 in Schrank and Vainstein (2010) and Figure 1 in Li and Xia (2022).

**Figure 1:**
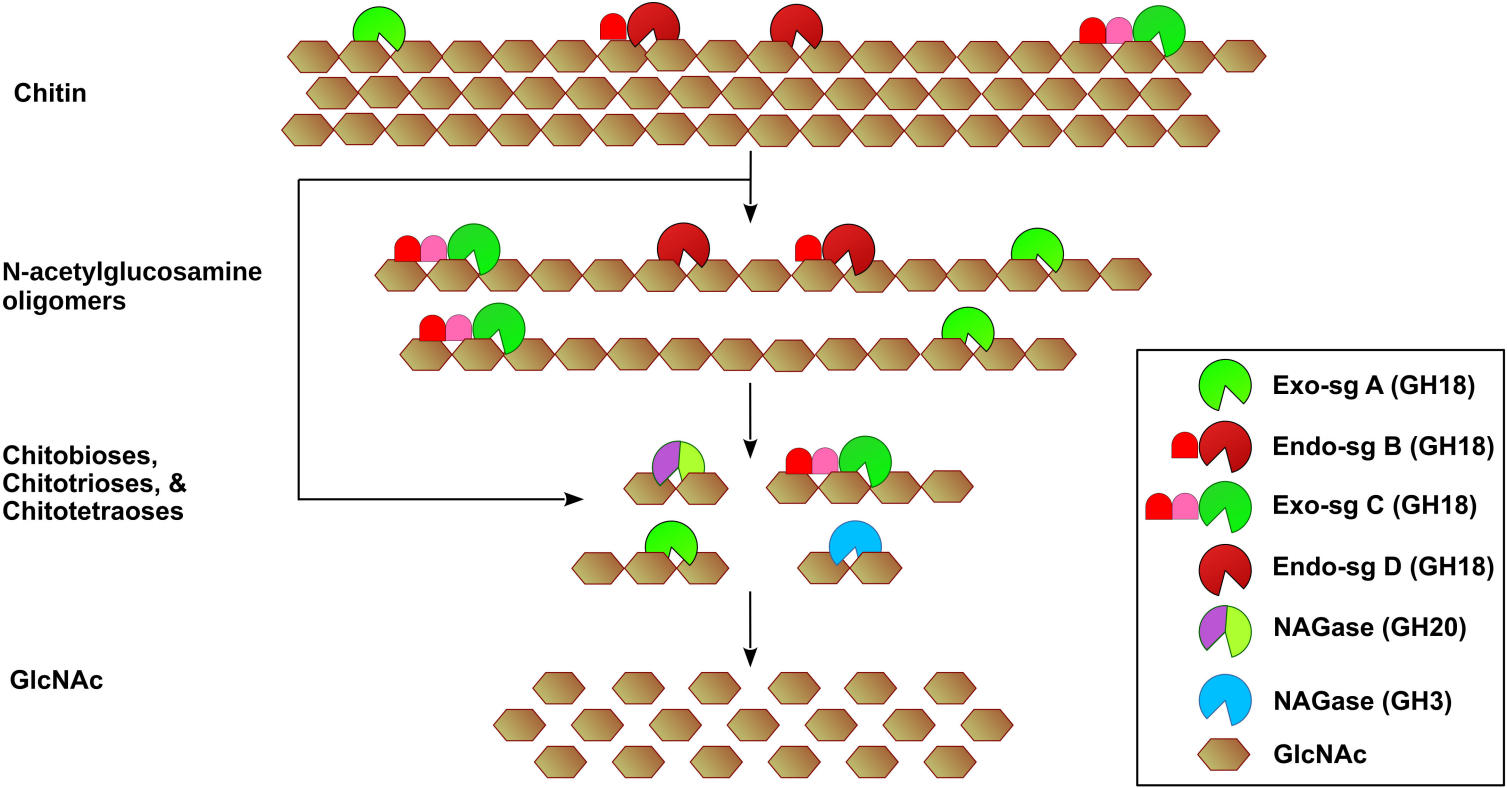
Schematic diagram for the hydrolysis of chitin by *M. anisopliae* enzymes. The endo-acting chitinases of subgroups B and D hydrolyze glycosidic bonds randomly, producing soluble short chains of GlcNAc oligomers (5–10 GlcNAc units). The subgroups A and C exo-chitinases act on chitin terminal ends, releasing chitobioses (containing 2 GlcNAc molecules), chitotrioses (containing 3 GlcNAc molecules), and chitotetraoses (containing 4 GlcNAc molecules). The hydrolysis products are further hydrolyzed to GlcNAc by exo-chitinases and β-N-acetylglucosaminidases (NAGases). Although subgroup A chitinases are predicted to exhibit exo-acting activity, CHIT42 displays an endo-acting activity. CHI30, which belongs to the endo-acting subgroup B, exhibits a dual endo- and exo-chitinase activity.

**Figure 2:**
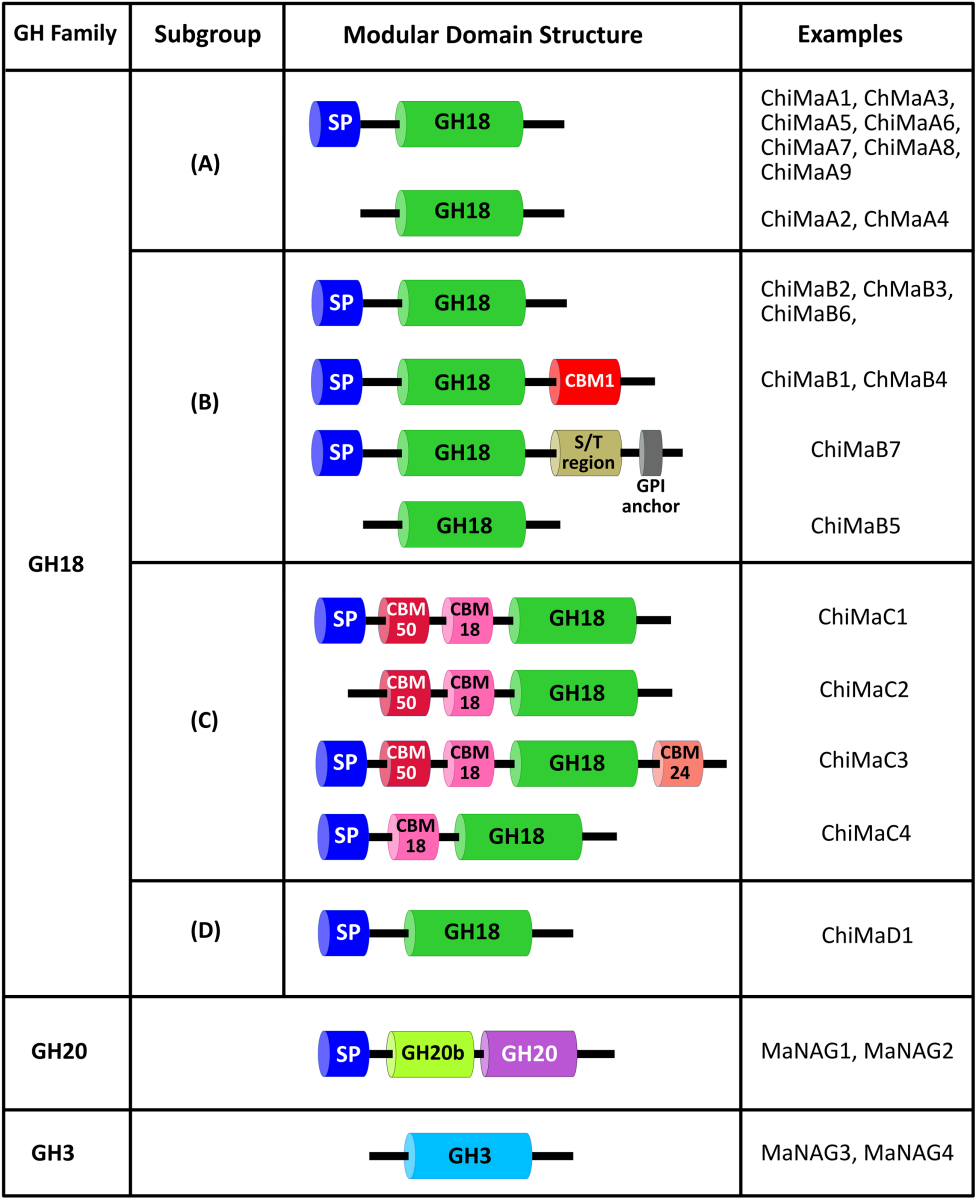
Domain architecture of chitinases and NAGase from *M. anisopliae*. SP, signal peptide; GH18, GH18 catalytic domain; GH20, GH20 catalytic domain; GH3, GH3 catalytic domain; GH20b, non-catalytic GH20b domain; CBM, carbohydrate-binding module; S/T region, Ser/Thr linker region; GPI anchor, GPI for anchoring protein to the plasma membrane.

The first genome sequence of *M. anisopliae* was published using the isolate ARSEF23 as a reference strain; however, this isolate was reclassified as *Metarhizium robertsii* (Gao et al., 2011). The complete genome sequence of *M. anisopliae* was repeated using the isolates E6 and Ma69 (Pattemore et al., 2014; Staats et al., 2014; Iwanicki et al., 2022). The genome size of *M. anisopliae* is about 38.5 Mb with 127 proteins that have no homologs in *M. robertsii* or *M. acridum* genome. The fungus produces 1620 secreted proteins, including an arsenal of proteases and chitinases that allow for cuticle penetration and host colonization (Pattemore et al., 2014; Staats et al., 2014). *M. anisopliae* chitinases are key players in fungal virulence against different stages of insect pests.

To understand their activity and specificity, chitinases from the entomopathogenic fungus *M. anisopliae* have been the subject of extensive studies that span biochemical characterization, structural analysis, and regulation of gene expression. However, comprehensive reviews that highlight these findings are lacking. The main objectives of this review are to (1) consolidate our current knowledge on chitin-hydrolyzing enzymes from *M. anisopliae*, including their glycoside hydrolase (GH) classification and domain architecture, and (2) identify conserved molecular mechanisms underlying chitinase gene expression using studies from other entomopathogenic fungi.

## 2 Chitin as a structural barrier in insect hosts

Insects have an external complex structure called the exoskeleton. The insect exoskeleton, which is also called the cuticle, is composed of two non-living layers: the epicuticle and the procuticle (Chen et al., 2008; Andersen, 2009; Doucet and Retnakaran, 2016; Vittori, 2024). The cuticle serves essential functions in insect support and protection. For example, it serves as an external framework that supports the body of insects and determines their shape. In addition, muscles are attached to the cuticle and thus aid in insects’ mobility. Furthermore, the exoskeleton operates as a barrier protecting insects from external environmental factors and physical damage (Gorb, 2001; Vincent, 2001; Hamid et al., 2013). The exoskeleton of insects generally contains 30% to 45% protein and 25% to 40% lipids. The chitin content in the exoskeleton varies among the species, ranging from 5 to 15% (Izadi et al., 2025).

Chitin is a linear polysaccharide of N-acetylglucosamine (*abbr.:* GlcNAc) repeating units that are linked by β-1,4-glycosidic bonds. Structurally, GlcNAc is similar to glucose but has an acetamide group at the C2 position instead of the hydroxyl group (Jones et al., 2020). Chitin exists in three allomorphic forms: α-chitin, β-chitin, and γ-chitin, which differ in chain orientation and hydrogen bonding patterns, resulting in distinct mechanical properties. α-chitin consists of antiparallel chains stabilized by intra- and intermolecular hydrogen bonds, resulting in greater stiffness. In contrast, the structure of β-chitin consists of parallel chains connected via weak intermolecular hydrogen bonds. β-chitin exhibits a reduced stiffness that provides more flexibility compared to α-chitin. Both α-chitin and β-chitin are commonly found in arthropods. The structure of γ-chitin exhibits a mixture of α- and β-chitin, and it is mainly found in fungi, yeasts, and insect cocoons. For a schematic representation of the three allomorphic forms of chitin, refer to Figure 1 in Mei et al. (2024) and Figure 4 in Kumirska et al. (2011). Partial deacetylation of chitin results in the production of chitosan, which is a linear polymer of D-glucosamine and N-acetyl-D-glucosamine (Al-Nasrawi et al., 2024; Abdulhameed et al., 2025).

**Figure 3:**
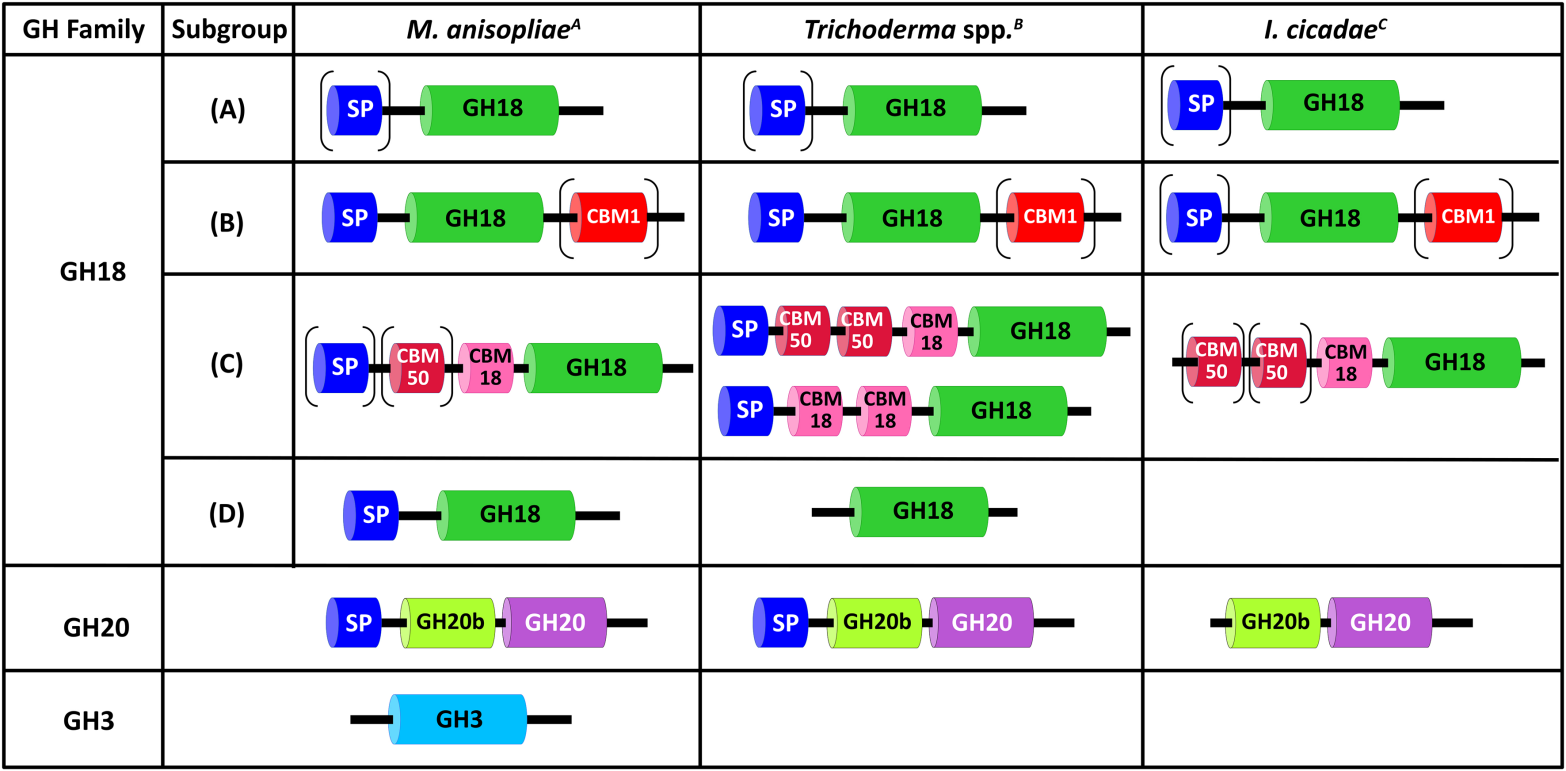
Comparison of the domain architecture of the chitinolytic enzymes from *M. anisopliae*, *Trichoderma* spp., and *Isaria cicadae*. SP: signal peptide, GH18: GH18 catalytic domain, GH20: GH20 catalytic domain, GH3: GH3 catalytic domain, GH20b: non-catalytic GH20b domain, CBM: carbohydrate-binding module. Data were collected from ^A^ (Junges et al., 2014; Oliveira et al., 2018), ^B^ (Seidl et al., 2005; López-Mondéjar et al., 2009; Häkkinen et al., 2012; Seidl-Seiboth et al., 2014), and ^C^ (Peng et al., 2020).

## 3 Hydrolysis of chitin by chitinases and β-N-acetylglucosaminidases from *M. anisopliae*

The degradation of chitin by fungal chitinolytic enzymes is a consecutive and coordinated multistep process that involves exo-chitinases, endo-chitinases, lytic polysaccharide monooxygenases (LPMOs), and β-N-acetylglucosaminidases (NAGases) (Busk and Lange, 2015). Chitinases also exhibit enzymatic activity against chitosan; however, such activity is strongly dependent on the degree of acetylation (Hartl et al., 2012). It should be mentioned that LPMOs will not be covered in this review because of a lack of sufficient data. In this review, the terms chitinolytic enzymes, chitin-degrading enzymes, and chitin-hydrolyzing enzymes are used interchangeably to refer to chitinases and NAGases.

The classification of chitinases can be based on either the mode of action or amino acid sequence similarities (Henrissat and Davies, 1997; Poria et al., 2021). The Nomenclature Committee of the International Union of Biochemistry and Molecular Biology (IUBMB) classifies chitinases based on their mode of action into endo-acting chitinases and exo-acting chitinases. Endo-acting chitinases (EC 3.2.1.14) cleave randomly along chitin internal chains, producing soluble N-acetylglucosamine oligomers (Poria et al., 2021). Exo-acting chitinases act on chitin terminal ends at the reducing end (EC 3.2.1.201) and the non-reducing end (EC 3.2.1.200), releasing chitobioses (contain two GlcNAc molecules), chitotrioses (contain three GlcNAc molecules), and chitotetraoses (contain four GlcNAc molecules). Chitin hydrolysis products are further cleaved by exo-chitinases and β-N-acetylglucosaminidases (NAGases) (EC 3.2.1.52) (Hartl et al., 2012; Muschiol et al., 2020; Jiang et al., 2025) (**Figure 1**).

However, the IUBMB classification does not provide insight into enzymes structural features or predict their 3D structures (Henrissat and Davies, 1997). The Carbohydrate-Active Enzymes database (CAZy, https://www.cazy.org/) has, therefore, developed a classification system based on amino acid sequence similarity, enabling the grouping of enzymes with conserved structures and providing insights into protein structure and mechanism (Henrissat and Davies, 1997).

The class glycoside hydrolases (GHs) contains enzymes with EC 3.2.1.- activity that hydrolyze glycosidic bonds (Henrissat and Davies, 1997; Talamantes et al., 2016). Under this classification system, endo- and exo-chitinases are placed in GH families 18 and 19, whereas β-N-acetylglucosaminidases are distributed in GH families 3, 20, and 84 (Oyeleye and Normi, 2018). Fungal chitinases are exclusively found in GH family 18. The only exception is NbChiA (a chitinase isolated from the fungus *Nosema bombycis*), which is the only known fungal chitinase that belongs to GH family 19 (Han et al., 2016).

*M. anisopliae* possesses a complex arsenal of chitin-degrading enzymes; however, only four chitinases have been functionally characterized (**Table 1**). CHIT42 is the first characterized chitinase from *M. anisopliae*. It is an endo-acting chitinase that is composed of 423 amino acid and has a molecular mass of 42 kDa. The enzyme contains a signal peptide for protein secretion (Bogo et al., 1998; Baratto et al., 2003). Despite its clear biochemical activity, constitutive expression of the *chit1* gene under the control of the *Aspergillus nidulans gpdA* promoter did not elevate virulence toward *Manduca sexta* caterpillars, suggesting that increased chitinase activity alone is insufficient to enhance pathogenicity (Screen et al., 2001). CHI2 is another chitinase that was characterized in *M. anisopliae*. The enzyme, which exhibits endochitinase activity, is composed of 419 amino acids and has a molecular mass of 42 kDa (Baratto et al., 2006; Boldo et al., 2009). In contrast to CHIT42, CHI2 appears to play a more direct role in virulence. Over-expression of the *chi2* gene in *M. anisopliae* results in a strain with elevated virulence against the cotton stainer bug (*Dysdercus peruvianus*). Deletion of the *chi2* gene in *M. anisopliae* reduced fungal virulence by 20% compared to the wild type strain (Boldo et al., 2009). These observations are consistent with broader trends in *Metarhizium* and *Beauveria*, where virulence differences often reflect variation in expression and deployment of secreted enzymes rather than their mere presence (Ortiz-Urquiza and Keyhani, 2013). Interestingly, two proteins are produced from the *chi2* gene via alternative splicing of the *chi2* transcripts (Boldo et al., 2010). CHIT30 is a 30 kDa chitinase from *M. anisopliae* that is encoded by the *chi3* gene and exhibits dual endo- and exochitinase activities (Pinto et al., 1997; da Silva et al., 2005). Deletion of the *chi3* gene reduced fungal virulence against cotton stainer bug *D. peruvianus*, suggesting a major role in cuticle degradation and host penetration. Interestingly, the secretion of CHIT30 is also induced by heat shock at 40°C, suggesting another role of CHIT30 in heat-shock adaptation (Staats et al., 2013). This multifunctional role aligns with observations in other hypocrealean fungi, including *Trichoderma* spp., where secreted chitinases contribute both to host interaction and environmental adaptation (Kubicek et al., 2011; Mukherjee et al., 2013). ChiMaD1 is the fourth chitinase to be characterized from *M. anisopliae*. The molecular characterization and virulence impact of ChMaD1 will be discussed below.

**Table 1:** The properties and virulence impacts of the characterized chitinases from *M. anisopliae*.

Enzyme (gene name)	GH18 family subgroup	Molecular weight (kDa)	Domain organization	Activity	Virulence impact
CHIT42 (*chit1*)	A	42	SP – GH18	Endo	• Overexpression did not alter virulence against *M. sexta* caterpillars.
CHI2 (*chi2*)	B	42	SP – GH18 – CBM1	Endo	• Deletion mutant exhibited reduced virulence against cotton stainer bug (*D. peruvianus*) • Over-expression elevated virulence against cotton stainer bug.
CHI30 (*chi3*)	B	30	SP – GH18	Endo & Exo	• Deletion mutant exhibited reduced fungal virulence against cotton stainer bug.
ChiMaD1	D	36.6	SP – GH18	Endo	• Deletion mutant exhibited higher virulence rates against the cattle tick (*R. microplus*); however, did not alter virulence against the mealworm larvae (*T. molitor*).

SP, signal peptide, GH18, GH18 catalytic domain, CBM1, Carbohydrates Binding Module 1, Endo, endo-chitinase, Exo, exo-chitinase.

### 3.1 GH18 family proteins

The glycoside hydrolase family 18 (GH18) includes fungal chitinases as well as chitinases from other organisms, such as bacteria, animals, and plants (reviewed in detail by Chen et al., 2020). In general, chitinases of the GH18 family possess a conserved catalytic domain that adopts a (β/α)8 TIM (triosephosphate isomerase) barrel fold. Additionally, the substrate-binding site of GH18 chitinases typically accommodates at least five sugar units, where cleavage occurs between the third and fourth units. Enzymes of the GH18 family are further subgrouped into class III and class V chitinases. These two classes differ in their mode of action and substrate binding groove, which influences substrate specificity and enzymatic function (Seidl, 2008; Hartl et al., 2012).

The domain architecture of GH family 18 chitinases comprises a combination of four functional regions: (1) a signal peptide that directs secretion of the protein outside the cell, (2) a GH18 catalytic domain that cleaves β-1,4 glycosidic bonds between GlcNAc units, (3) a carbohydrate (or chitin)-binding module (CBM) that facilitates binding and hydrolysis of insoluble chitin, and (4) a serine (Ser)/threonine (Thr)-rich linker. The biological function of the linker is not fully understood; however, it has been proposed that the Ser/Thr-rich linker enhances the enzymatic activity against insoluble substrates and protects chitinases from proteolytic degradation, particularly by extracellular proteases (González et al., 2012; Huang et al., 2012). In addition, the Ser/Thr-rich linker may contribute to proper protein folding and has been implicated in glycosylphosphatidylinositol (GPI) anchoring of chitinases to the plasma membrane (Junges et al., 2014).

Based on the comparative analysis of 18 chitinases from the filamentous fungus *Trichoderma reesei*, fungal GH18 chitinases have been classified into three subgroups designated A, B, and C (Seidl et al., 2005). This classification system is based on a combination of protein domain architecture, enzymatic mode of action (endo- or exo-acting), and the structural and physiochemical properties of the substrate-binding cleft (Hartl et al., 2012). Subgroups A and B show homology to class V and class III, respectively, of the GH18 family. In contrast, subgroup C comprises a novel class of high molecular weight chitinases. Interestingly, one chitinase, designated Chi18-15, did not exhibit sequence similarity to any of the three fungal subgroups (Seidl et al., 2005). Nevertheless, Seidl et al. (2005) did not assign Chi18–15 to a distinct subgroup.

*In silico* analysis of the *M. anisopliae* genome revealed the presence of 21 putative chitinases, including the three characterized chitinases (CHIT42, CHI2, and CHI30). Based on sequence homology, these enzymes were categorized into the three fungal subgroups: A, B, and C (Junges et al., 2014). The distribution of enzymes across the three subgroups was as follows: nine enzymes were grouped in subgroup A and were designated ChiMaA1 to ChiMaA9, seven enzymes were grouped in subgroup B and were designated ChiMaB1 to ChiMaB7, and four enzymes were grouped in subgroup C and were designated ChiMaC1 to ChiMaC4. In addition, Junges et al. (2014) assigned a chitinase that is homologous to the *T. reesei* Chi18–15 to a novel subgroup D and was designated ChiMaD1 (**Table 2**). As anticipated, the analysis of *M. anisopliae* genome did not identify any GH19 chitinases.

**Table 2:** Chitinases and β-N-acetylglucosaminidases identified in *M. anisopliae*.

GH family	GH18 subgroup	Protein	Protein accession number	Published locus tag	Notes
GH18	A	ChiMaA1	KFG78916	MANI_007345	Also designated CHIT42 (gene: *chit1*).
		ChiMaA2	KFG82581	MANI_003521	
		ChiMaA3	KFG85343	MANI_022967	
		ChiMaA4	KFG87333	MANI_012760	
		ChiMaA5	KFG82099	MANI_005739	
		ChiMaA6	KFG82955	MANI_029841	
		ChiMaA7	KFG84119	MANI_000730	
		ChiMaA8	KFG79492	MANI_021851	
		ChiMaA9	KFG81193	MANI_006991	
	B	ChiMaB1	KFG78638	MANI_002801	Also designated CHI2 (gene: *chi2*).
		ChiMaB2	KFG84275	MANI_115994 ^A^	Also designated CHI30 (gene: *chi3*).
		ChiMaB3	KFG78862	MANI_026679	
		ChiMaB4	KFG77924	MANI_004417	
		ChiMaB5	KFG77460	MANI_021602	
		ChiMaB6	KFG77554	MANI_018482	
		ChiMaB7	KFG80416	MANI_012994	
	C	ChiMaC1	KFG83700	MANI_023684	
		ChiMaC2	KFG80074	MANI_120160 ^A^	
		ChiMaC3	KFG84782	MANI_115484 ^A^	
		ChiMaC4	KFG84211	MANI_116045 ^A^	
	D	ChiMaD1	KFG86059	MANI_018860	
GH20	N/A ^B^	MaNAG1	KFG80340	MANI_010908	
	N/A ^B^	MaNAG2	KFG85702	MANI_029504	
GH3	N/A ^B^	MaNAG3	KFG78085	MANI_122030	
	N/A ^B^	MaNAG4	Not found	MANI_128875	

^A^ Published locus tag was corrected and updated in this table.

^B^ NA, not applicable.

#### 3.1.1 Fungal subgroup A chitinases

Fungal subgroup A chitinases constitute the predominant subgroup of fungal GH18 chitinases and are conserved across all fungal genomes. These enzymes typically exhibit a low molecular weight ranging from 40–50 kDa. The substrate-binding cleft of subgroup A enzymes is deep and narrow. The majority of subgroup A chitinases are composed of an N-terminal signal peptide followed by a GH18 catalytic domain. Despite this conserved domain architecture, some subgroup A chitinases lack the signal peptide and are therefore predicted to remain intracellularly or to be secreted via unconventional protein secretion pathways (Seidl, 2008; Ding et al., 2012). In *M. anisopliae*, seven subgroup A chitinases exhibit an N-terminal signal peptide for secretion outside the cell, whereas the remaining two enzymes (ChiMaA2 and ChiMaA4) lack a signal peptide (Junges et al., 2014). The well-characterized CHIT42 (ChiMaA1) of *M. anisopliae* belongs to subgroup A and contains both an N-terminal signal peptide and a GH18 catalytic domain consistent with the subgroup A architecture (**Figure 2**).

#### 3.1.2 Fungal subgroup B chitinases

The majority of subgroup B chitinases are low molecular weight enzymes, typically ranging from 30–50 kDa. In contrast to subgroup A, the substrate-binding cleft is shallow and open. The domain architecture of subgroup B chitinases consists of an N-terminal signal peptide, followed by a GH18 catalytic domain. In addition, subgroup B chitinases frequently contain a C-terminal CBM module (Hartl et al., 2012). Some subgroup B chitinases harbor additional domains such as a Ser/Thr-rich domain and a C-terminal glycosylphosphatidylinositol (GPI)-anchoring motif for attaching the protein to the plasma membrane (Takaya et al., 1998; Yamazaki et al., 2008).

In *M. anisopliae*, six subgroup B chitinases exhibit a GH18 catalytic domain and an N-terminal signal peptide. In contrast, ChiMaB5 is the only subgroup B chitinase that lacks the signal peptide. Both ChiMaB1 and ChiMaB4 have an additional C-terminal CBM1 for strong attachment to insoluble chitin. Interestingly, ChiMaB7 displays a domain-rich architecture comprising the signal peptide, GH18 catalytic domain, and a C-terminus Ser/Thr-rich region coupled with a GPI anchoring motif (Junges et al., 2014). Both CHI2 and CHI30 of *M. anisopliae* belong to subgroup B of GH18 protein. CHI2 comprises an N-terminal signal peptide, a GH18 catalytic domain, and a C-terminal CBM (Boldo et al., 2009). CHIT30 exhibits a similar domain architecture but lacks the C-terminal CBM module (**Figure 2**) (Junges et al., 2014).

#### 3.1.3 Fungal subgroup C chitinases

Fungal subgroup C chitinases share several structural and functional features with subgroup A enzymes. For example, subgroup A and C enzymes are both exo-acting enzymes that belong to class V chitinases and have a deep and narrow substrate-binding groove. Nevertheless, subgroup C chitinases have several features that distinguish them from subgroups A and B. First, subgroup C enzymes are high molecular weight proteins (140–170 kDa). In addition, the domain organization of subgroup C chitinases contains a CBM 18 domain and a CBM 50 domain (also known as LysMs) for binding insoluble chitin. Both CBM 18 and CBM 50 are located at the N-terminal region of subgroup C (Seidl, 2008; Rajput et al., 2022).

*M. anisopliae* chitinases from the subgroup C exhibit multiple CBM modules in addition to a GH18 catalytic domain and an N-terminal signal peptide. For example, the amino acid sequence of ChiMaC1 and ChiMaC2 harbor two CBMs, i.e. CBM18 and CBM50, whereas ChiMaC3 has three CBMs, i.e. CBM18, CBM24, and CBM50. The only exception to this is ChiMaC4, which displays only one CBM, i.e. CBM18 (Junges et al., 2014). Interestingly, ChiMaC2 lacks the signal peptide region (**Figure 2**), suggesting that this enzyme is a cytoplasmic enzyme, which functions in fungal cell wall remodeling. It is also possible that ChiMaC2 is secreted via unconventional protein secretion pathways (Ding et al., 2012).

#### 3.1.4 Fungal subgroup D chitinases

ChiMaD1 chitinase is a homolog of *T. reesei* chi18-15. ChiMaD1 orthologs exhibit endochitinase activity and contain a GH18 catalytic domain that is similar to the chitinase J (ChiJ) from *Streptomyces* species (Viterbo et al., 2001; Seidl et al., 2005; Ubhayasekera and Karlsson, 2012). The protein domain architecture is composed of an N-terminal signal peptide and a GH18 catalytic domain of bacterial origin (**Figure 2**) (Junges et al., 2014).

Deletion of ChMaD1-encoding gene in *M. anisopliae* did not affect the susceptibility of the cell wall to stresses or reduce secreted chitinases. The chMaD1-deletion mutant exhibits higher virulence rates against the cattle tick (*Rhipicephalus microplus*), but, surprisingly, displayed virulence rates similar to the wild type strain in the mealworm larvae (*Tenebrio molitor*) (Sbaraini et al., 2021). This suggests that cuticle variability across different species can induce the expression of different sets of chitinase genes.

### 3.2 GH20 and GH3 β-N-acetylglucosaminidases

β-N-acetylglucosaminidases (β-N-acetylhexosaminidases, NAGases, EC 3.2.1.52) are enzymes that catalyze the breakdown of chitobioses to GlcNAc monomers (Intra et al., 2008). NAGases are grouped into three GH families: GH3, GH20, and GH84. Fungal NAGases are commonly found in the GH20 family (Oliveira et al., 2018). Although the majority of GH3 NAGases are found in prokaryotes, a fungal GH3 NAGase was isolated and characterized from *Rhizomucor miehei* (Yang et al., 2014). Additionally, the genome of *M. anisopliae* harbors two NAGases from GH3 family. Therefore, only GH3 and GH20 NAGases will be covered in this section.

The characteristic domain architecture for GH20 proteins comprises a catalytic GH20 domain with an α/β TIM-barrel preceded by an N-terminal non-catalytic GH20b domain, which is required for protein expression and stabilization (**Figure 2**) (Liu et al., 2012; Val-Cid et al., 2015; Li et al., 2022b).

GH3 family enzymes exhibit a broad range of enzyme activities, such as β-D-glucosidases, α-L-arabinofuranosidases, β-D-xylopyranosidases, and N-acetyl-β-D-glucosaminidases (NAGases) (Macdonald et al., 2015). GH3 NAGases are considered single-domain enzymes because chitobiose hydrolysis is carried out exclusively by the N-terminal domain, which harbors the general acid/base catalytic residue and the catalytic nucleophile residue (Bacik et al., 2012; Macdonald et al., 2015). The presence of the general acid/base catalytic residue and the catalytic nucleophile residue in the GH3 catalytic domain is a distinctive feature for the identification of GH3 NAGases. In *T. reesei*, a protein, designated NAG3, with a GH3 NAGase domain was identified and thought to be the first *T. reesei* GH3 NAGase (Kappel et al., 2016). However, this protein lacks the catalytic residues responsible for the cleavage of amino sugar substrates, suggesting that it might function as a GlcNAc signal sensor/transducer. The protein was re-designated NGS1 (Ullah et al., 2025).

In an early study, a 110 kDa enzyme with NAGase activity was purified from *M. anisopliae*. The enzyme hydrolyzes GlcNAc dimers, trimers, and tetramers to GlcNAc monomers. The enzyme was feedback inhibited by the accumulation of GlcNAc (St. Leger et al., 1991). Nevertheless, the gene encoding for this enzyme was not identified.

Genome-wide screening revealed the presence of four NAGase enzymes in the genome of *M. anisopliae*. Two enzymes, MaNAG1 and MaNAG2, belong to GH20 NAGases and the other two, MaNAG3 and MaNAG4, belong to GH3 NAGases (**Figure 2**) (Oliveira et al., 2018). The molecular masses of MaNAG1 and MaNAG2 are ~67 kDa and ~61.5 kDa, respectively. The putative GH20 NAGases exhibit the characteristic GH20 domain architecture and an N-terminal signal peptide for secretion. MaNAG3 and MaNAG4 have a predicted molecular mass of ~99 and ~60.5 kDa, respectively. Bioinformatic analysis of MaNAG3 and MaNAG4 reveals the presence of the typical GH3 catalytic domain that contains the catalytic residues (Oliveira et al., 2018). The domain composition of MaNAG1–4 is summarized in **Figure 2** of this review. The expression profiles were described by others (Oliveira et al., 2018).

In contrast to chitinases, which are present in high redundancy within fungal genomes, NAGases are encoded by one or a few genes. Nevertheless, NAGases play important roles during *M. anisopliae* cell wall extension, branching, and degradation (Oliveira et al., 2018). In addition, NAGases play an important role in metabolizing chitin as the sole carbon source. The deletion of NAGase-encoding genes, *nag1* and *nag2*, in the mycoparasitic fungus *Trichoderma atroviride* abolished its ability to grow on chitin as the sole carbon source, highlighting the significance of NAGases in metabolizing chitin. Nevertheless, the deletion of NAGase-encoding genes did not impact fungal virulence, suggesting a minor role for chitin as a carbon source during parasitism (López-Mondéjar et al., 2009).

## 4 The biological functions of chitinases during pathogenesis and saprophytic growth

Entomopathogenic fungi have a complex life cycle that involves a parasitic stage on different arthropods, and a saprophytic stage in the soil or on infected cadavers (Gortari and Hours, 2008; Chen et al., 2020; St. Leger and Wang, 2020). Chitinases are produced during all stages of the fungal life cycle. Besides degrading exogenous chitin, chitinases are also key enzymes in fungal cell wall remodeling (Gruber et al., 2011). The number of these enzymes varies with the trophic relationship. For example, the genomes of the mycoparasitic fungi *T. atroviride* and *T. virens* harbor a greater number of chitinase-encoding genes than those of entomopathogenic and saprophytic fungi (**Table 3**). In contrast, the number of these genes in the entomopathogenic fungi is approximately equal to or lower than that in the saprophytic fungus *T. reesei*. Interestingly, the entomopathogenic fungus *B. bassiana* has more NAGase-encoding genes (5 genes) than other fungi (**Table 3**).

**Table 3:** The distribution of chitin-hydrolyzing enzymes in entomopathogenic, mycoparasitic, and saprophytic fungi.

Enzyme	Entomopathogenic					Mycoparasitic		Saprophytic
	*M. anisopliae ^A^*	*M. robertsii ^B^*	*M. acridum ^B^*	*B. bassiana ^B^*	*I. cicadae ^C^*	*T. atroviride ^D^*	*T. virens ^D^*	*T. reesei ^D^*
Chi-sg A	9	8	7	8	7	7	8	7
Chi-sg B	7	7	5	4	7	11	11	7
Chi-sg C	4	6	2	8	3	9	15	4
Chi-sg D	1	ND*^E^*	ND*^E^*	ND*^E^*	ND*^E^*	1	1	1
Total Chitinases	21	21	14	20	17	28	35	19
GH20 NAGase	2	2	2	5	1	2	2	2
GH3 NAGase	2	ND*^F^*	ND*^F^*	ND*^F^*	ND*^F^*	ND*^F^*	ND*^F^*	ND*^F^*
Total	25	23	16	25	18	29	36	21

Data were compiled from ^A^ (Junges et al., 2014; Oliveira et al., 2018); ^B^ (Xiao et al., 2012), ^C^ (Peng et al., 2020), and ^D^ (Seidl-Seiboth et al., 2014).

Chi-sg: Chitinase subgroup.

ND: Not Determined.

^E^ Chitinases that belong to the subgroup D were not identified in the original manuscripts. However, when we ran BLAST analysis using ChiMaD1 chitinase (Accession Number KFG86059) as query sequence, we identified orthologs in *M. robertsii* (Accession Number XP_007824504.1, similarity 96%), *M. acridum* (Accession Number XP_007808545.1, similarity 89.6%), *B. bassiana* (Accession Number XP_008599636.1, similarity 80%), and *I. cicadae* (Accession Number QDJ94322.1, similarity 78%).

^F^ NAGases that belong to the GH3 family were not identified in the original manuscripts. However, BLAST analysis using MaNAG3 (Accession Number KFG78085) as query sequence identified orthologs in *M. robertsii* (Accession Number XP_007818395.2, similarity 99.5%), *M. acridum* (Accession Number XP_065978122.1, similarity 95%), *B. bassiana* (Accession Number XP_008598947.1, similarity 77.5%), *T. atroviride* (Accession Number WOD46308.1, similarity 77.5%), *T. virens* (Accession Number XP_013958055.1, similarity 78%), and *T. reesei* (Accession Number XP_006966911.1, similarity 78%). Interestingly, MaNAG3 did not exhibit significant similarity to any protein encoded by the *I. cicadae* genome.

Chitinases exhibit distinct biological roles during the different stages of the fungal life cycle. Entomopathogenic fungi secrete an arsenal of chitinases that hydrolyzes the cuticle, facilitating host penetration and colonization. Inside the hemocoel, they metabolize host nutrients while exhibiting limited dependence on less−preferred carbon sources, including chitin (Gruber et al., 2011). Once the host nutrients are depleted, the entomopathogenic fungi switch to the saprophytic stage, growing on the cadavers of insects. During this stage, they have a higher capacity to metabolize the less-preferred carbon sources. This suggests a distinction between chitinase-mediated virulence and chitin metabolism. This is supported by the fact that the large arsenal of chitin-degrading enzymes in mycoparasitic fungi (**Table 3**) did not account for a good metabolism of chitin or its partially acetylated form, chitosan. In contrast, the entomopathogenic fungus *B. bassiana* exhibited a better growth on chitin despite having a fewer chitinase genes (Palma-Guerrero et al., 2008; Gruber et al., 2011). Furthermore, the deletion of NAGase-encoding genes in *T. atroviride* results in strains with abolished growth on chitin as the sole carbon source; nevertheless, the mutants retained full virulence (López-Mondéjar et al., 2009). Because of the overlapping functions of chitinases, it is challenging to precisely delineate their relative contributions to each lifestyle (Ortiz-Urquiza and Keyhani, 2016).

Differences have also been noted between parasitic and saprophytic fungi with respect to the GH domain architecture. The genomes of the mycoparasitic fungi harbor a larger set of CBMs than those of the saprophytic fungi. These modules facilitate stronger attachment of GH proteins to insoluble substrates (Kubicek et al., 2011; Xiao et al., 2012; St. Leger and Wang, 2020). In *M. anisopliae*, the GH18 subgroup C chitinases have CBM-rich domain architecture (**Figure 2**), suggesting a major role for these proteins during pathogenicity.

The chitinolytic enzymes of the entomopathogenic fungi *M. anisopliae* and *Isaria cicadae*, and the mycoparasitic fungi belonging to *Trichoderma* spp. exhibit a conserved domain architecture in subgroups A and B of GH18 chitinases (**Figure 3**). Subgroup A enzymes contain a GH18 catalytic domain and frequently have a signal peptide at the N-terminus; however, these enzymes lack CBM modules. Subgroups B share a homologous domain organization that comprises a signal peptide followed by a GH18 module. Some subgroup B chitinases might also have a CBM1 module at the C-terminus (**Figure 3**). Interestingly, only a third of the subgroup B chitinases from *I. cicadae* have a signal peptide (Peng et al., 2020). In *Trichoderma* spp., subgroup D chitinase lacks signal peptide at the N-terminus. Peng et al. (2020) did not report a subgroup D chitinase in *I. cicadae*. However, we identified a homolog of subgroup D chitinase when we ran a BLAST analysis (**Table 3**). Subgroup C chitinases display a divergent domain organization across all three fungi (**Figure 3**). Although they play a key role in the degradation of the exogenous chitin, surprisingly, the subgroup C chitinases from *I. cicadae* lack the signal peptide. The domain architecture of GH20 NAGases is conserved except for the *I. cicadae* homolog, which lacks the signal peptide (**Figure 3**). The lack of the signal peptide in several chitinolytic enzymes from *I. cicadae* suggests different biological roles or that the proteins are secreted via unconventional pathways (Ding et al., 2012). Interestingly, homologs of GH3 NAGases from *M. anisopliae* have not been identified in *Trichoderma* spp. or *I. cicadae*. However, our BLAST analysis identified GH3 NAGase proteins in these fungi (**Table 3**). For reviews on the contributions of chitinase diversity to fungal virulence, we recommend the reader to refer to Hartl et al. (2012), Gruber and Seidl-Seiboth (2012), and Ferreira and Soares (2023).

## 5 Regulation of gene expression of chitin-degrading enzymes in *M. anisopliae*

Chitinase expression has been shown to correlate with virulence in entomopathogenic fungi. Highly virulent *M. anisoplia*e strains produced significant amounts of chitinases in comparison with low-virulence strains (Bhanu Prakash et al., 2012). The expression of *M. anisoplia*e chitinases is induced by different substrates such as insect cuticle, chitin, and GlcNAc (Bogo et al., 1998; Krieger de Moraes et al., 2003; Barreto et al., 2004; Santi et al., 2010b; Beys-da-Silva et al., 2014; Rustiguel et al., 2016). Unmasking chitin is a prerequisite for the induction of chitinase expression. Chitinase activities of entomopathogenic fungi, including *M. anisopliae*, are detected at low levels on the cuticle surface during the initial penetration step. However, chitinase levels increase once chitin becomes accessible following the removal of masking proteins through proteolytic hydrolysis (St. Leger et al., 1996). Because of the dual nature of *M. anisoplia*e as an insect pathogen and a soil saprophyte, chitinases are secreted in response to cuticles derived from living as well as dead insects. For example, the expression of chitinase genes was induced during cuticle penetration of the living *Helicoverpa armigera* insects, as well as during saprophytic growth on the cuticle of dead *Spodoptera litura* (Bhanu Prakash et al., 2012). In contrast, chitinase and NAGase activities are repressed in the presence of elevated concentrations of GlcNAc, suggesting a feedback inhibition mechanism regulating the expression of these genes (St. Leger et al., 1991; Krieger de Moraes et al., 2003; Barreto et al., 2004).

The spatiotemporal expression pattern of chitinases in *M. anisoplia*e varies with the substrate. Gene expression analyses deciphered the spatiotemporal expression pattern of putative GH18 chitinases, and GH3 and GH20 NAGases in *M. anisopliae*. Autolysis of mycelia induced the highest number of chitinases. Additionally, chitin and GlcNAc induced the highest expression levels of subgroups A and B chitinases in mycelia. In contrast, subgroup C chitinases displayed significantly lower expression levels than subgroups A and B under the same induction conditions. Fungal conidia, appressoria, and blastospores displayed lower expression levels than mycelia (Junges et al., 2014). Variability in gene expression levels was also noted in GH3 and GH20 NAGases. In mycelia, chitin induced various expression levels of NAGase-encoding genes. MaNAG1 and MaNAG4 showed the highest expression levels, reaching ~50-fold and ~13-fold, respectively. Interestingly, the expression of MaNAG2 and MaNAG4 genes was constantly detected in the appressoria in the absence of chitin or cuticle, and in mycelia after challenging with chitin, suggesting a major role in host cuticle penetration. MaNAG3 seems to have a dual role in fungal pathobiology and cell wall remodeling during hyphal growth. This can be attributed to the fact that the MaNAG3-encoding gene was constitutively expressed in conidia and appressoria; however, was induced in mycelia after challenging with chitin and GlcNAc (Oliveira et al., 2018). The spatiotemporal regulation of chitinases in other fungi has been reviewed by Langner and Göhre (2016).

The expression of chitinases is also triggered by inducers other than GlcNAc-containing substrates. For example, the expression of *M. anisoplia*e *chi3* (ChiMaB2) gene is regulated in response to heat-shock stress conditions (at 42°C for 1 h). It is therefore presumed that the *chi3* gene is a component of the fungal adaptation mechanism to heat-shock stress conditions (Staats et al., 2013).

Interestingly, infection with mycoviruses is another factor that affects chitinase activity. Mycoviruses are viruses that infect fungi, reducing their growth and virulence. Virus-infected *Metarhizium majus* and *B. bassiana* strains displayed an attenuated virulence and cuticular penetration (Wang et al., 2023; Zhang et al., 2023). Similarly, viral infection of *M. anisopliae* resulted in a significant reduction in endochitinase levels but not in N-acetylglucosaminidase levels (de la Paz Giménez-Pecci et al., 2002). However, such infection outcomes were found to be inconsistent across different studies. For example, Perinotto et al. (2014) showed that the virus-infected *M. anisopliae* strains exhibited higher virulence levels against *Rhipicephalus microplus* ticks and produced a significant amount of chitinases and proteases. These findings were supported by another study, which found that *M. anisopliae* and *M. pingshaense* mycovirus-infected strains exhibited elevated levels of virulence against the lepidopteran larvae of diamondback moth (*Plutella xylostella*) and the fall armyworm (*Spodoptera frugiperda*) (Guo et al., 2024). For more information on mycoviruses in entomopathogenic fungi, we recommend the reader to refer to recent reviews (Hough et al., 2023; Camargo et al., 2025).

**Figure 4:**
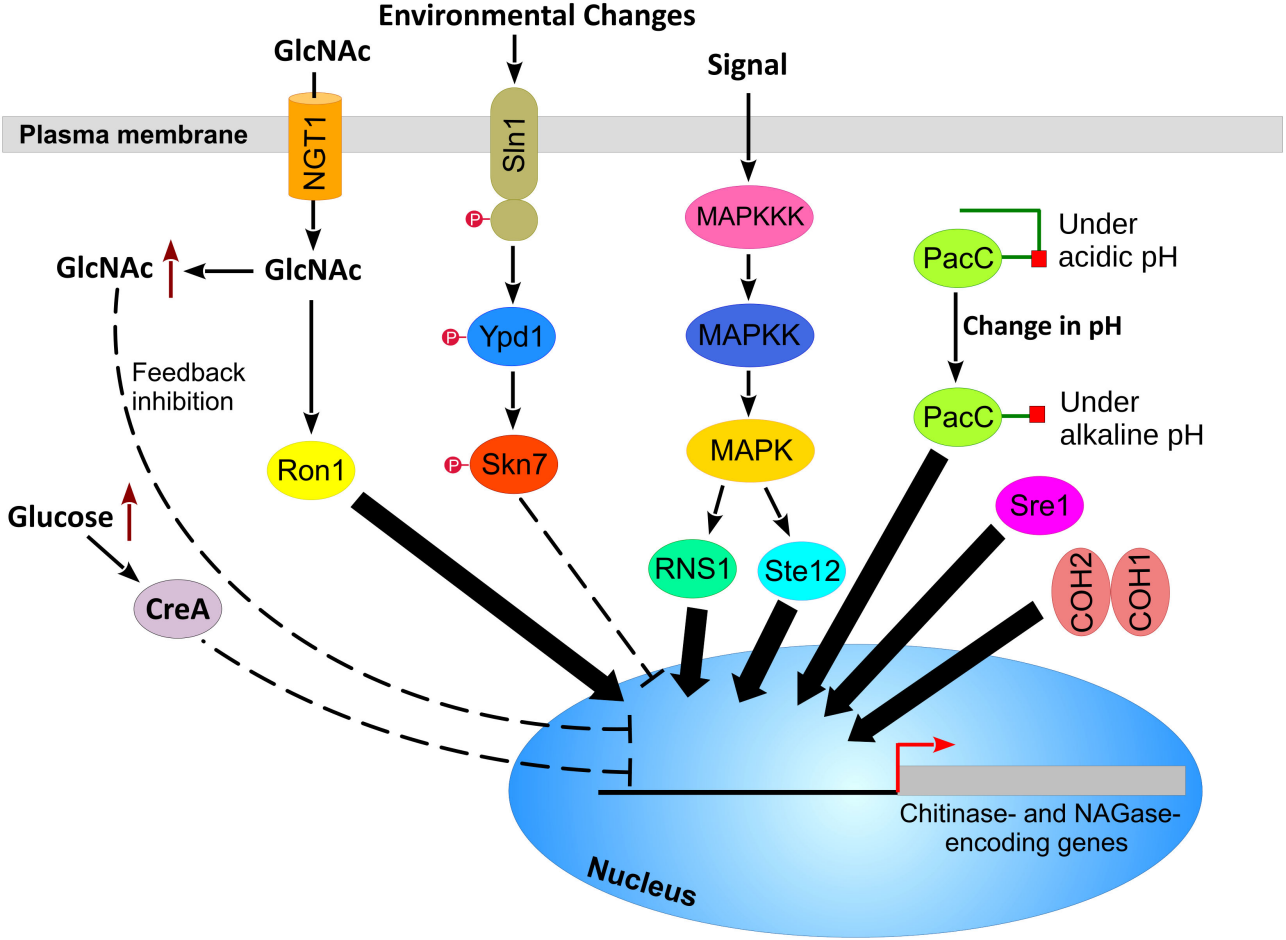
Putative regulatory pathways and transcription factors mediating the expression of chitin-hydrolyzing enzymes in *M. anisopliae*. GlcNAc is imported to the cytoplasm via the N‐acetylglucosamine transporter NGT1, causing the activation of the Ron1 transcription factor. The accumulation of GlcNAc in the cell causes a feedback inhibition of chitinolytic enzyme activities. The accumulation of glucose in the cell activates the carbon catabolite repressor CreA, which represses the activity of chitinases and NAGases. Changes in environmental conditions activate the two-component system sensor histidine kinase (Sln1), which activates downstream proteins. The activation of the Skn7 transcription factor represses the chitinase genes. Fus3 MAPK activates the RNS1 and Ste12 transcription factors, which up-regulate the expression of chitinolytic enzymes. Alkaline pH activates the PacC protein, which up-regulates the expression of chitinases and NAGases. In addition, Sre1 and COH2 are transcription factors that regulate the expression of chitinases in *M. anisopliae*.

*M. anisopliae* reacts to environmental changes via several sensory systems and a network of transcription factors. Screening of *M. anisopliae* genome identified 161 protein kinases and 510 putative transcription factors (Gao et al., 2011). However, the role of these proteins was not investigated in *M. anisopliae*. In this section, we will integrate key findings from other entomopathogenic fungi to identify conserved molecular mechanisms regulating gene expression of chitinases and NAGases in *M. anisopliae* (**Figure 4**).

The two-component system (TCS) is a key sensory system in fungi. The TCS system is a signal transduction pathway that consists of a membrane-bound sensor histidine kinase (Sln1) and two response regulator proteins, designated Ssk1 and Skn7 (Schaller et al., 2011). The histidine kinase (Sln1) is a hybrid protein that contains two domains: the kinase domain and the receiver domain. Sln1 senses environmental changes, and stimulates autophosphorylation of the kinase domain, which passes the phosphate group to the receiver domain of Sln1. Orthologs of Sln1 are involved in adaptation to different stress conditions (Catlett et al., 2003). In the entomopathogenic fungus *Metarhizium rileyi*, Sln1 is a key sensor of growth stress (Song et al., 2013, Song et al., 2015). Ypd1 is the next protein in the TCS. Ypd1 receives the phosphate group from Sln1 and transfers it to the downstream response regulators Ssk1 and Skn7. The later protein, Skn7, is a transcription factor that upregulates the expression of stress response genes and cell wall integrity in fungi (Fassler and West, 2011). The role of Skn7 in entomopathogenicity was analyzed in *Metarhizium robertsii*. Deletion of the *skn7* gene in *M. robertsii* induced cell autolysis and the activation of extracellular chitinases and proteases, which implies that Skn7 functions as a repressor of chitinase and protease gene expression. In addition, bioinformatic analysis revealed the presence of the Skn7 binding site 5′-GGC(C/G)(A/G)-3′ in the promoter region of two chitinases (Shang et al., 2015). The homologs of TCS proteins and the two chitinases are conserved in the genome of *M. anisopliae* (**Tables 4** and **5**). This suggests similar sensory and regulatory mechanisms in both fungi (**Figure 4**).

**Table 4:** The conservation of the regulatory mechanisms that control the expression of chitinases and NAGases in *M. anisopliae*.

Regulator/Transporter	Fungus	Protein accession number	Accession number of *M. anisopliae* homolog	Similarity (%)
Sln1	*M. rileyi*	AJF23735	KFG80059	71.1
Ypd1	*M. robertsii*	XP_007821550	KFG78655	98.7
Skn7	*M. robertsii*	XP_007820838	KFG82322	95.8
PacC	*M. robertsii*	XP_007822227	KFG86225	99.7
Ron1 (syn. PhoG or Vib1)	*B. bassiana*	XP_008598949	KFG78087	44.0
Sre1	*C. rosea*	CAG9943772	*KFG78094 ^A^	44.6
NGT1	*T. reesei*	XP_006968202	KFG79873	42.0
CreA	*M. acridum*	QCQ29101	CAA71314 ^B^	87.8
Fus3 MAPK	*M. robertsii*	XP_007820790	KFG82374	100
Hog1 MAPK	*M. robertsii*	XP_007821315	KFG87113	100
Slt2 MAPK	*M. robertsii*	XP_007819370	KFG81381	99.5
Ime2 MAPK	*M. robertsii*	XP_007821592	KFG84966	99.2
Ste7 MAPK	*M. robertsii*	XP_007820610	KFG80275	100
Pbs2 MAPK	*M. robertsii*	XP_007817045	KFG86447	97.5
Mkk MAPK	*M. robertsii*	XP_007822102	KFG77613	98.9
Ste11 MAPK	*M. robertsii*	XP_007819718	KFG80809	99.6
Bck1 MAPK	*M. robertsii*	XP_007820870	KFG82289	99.4
Ssk2 MAPK	*M. robertsii*	XP_007820479	KFG87534	98.9
RNS1	*M. robertsii*	XP_007823070	KFG85359	97.6
Ste12	*M. rileyi*	TWU73687	KFG86114	85.9
COH1	*M. robertsii*	XP_007825009	KFG84043	97.7
COH2	*M. robertsii*	XP_007824027	KFG84480	92.0

^A^ This enzyme is annotated as dihydrodipicolinate synthase; however, it exhibits sequence similarity to HMS1 transcription factor in *Saccharomyces cerevisiae*. Both Sre1 and HMS1 have the same Conserved Domains Database (CDD) number: cd11399.

^B^ BLAST analysis did not reveal any CreA homologs in E6 strain. Nevertheless, CreA homologs were found in other *M. anisopliae* strains, which suggests that the genome of E6 strain still requires improvement in gene annotation.

**Table 5:** Detected chitinases and NAGases and their cognate regulators in *M. robertsii*, and the homologs of these chitinases and NAGases in *M. anisopliae*.

Enzyme locus in *M. robertsii* (accession number)	Accession number of *M. anisopliae* homolog (protein name)	Similarity %	Regulators of this enzyme ^A^
Subgroup A chitinases			
MAA_02685 (XP_007818874)	KFG78916 (ChiMaA1)	99.0	PacC ↑
MAA_04700 (XP_007820987)	KFG87333 (ChiMaA4)	99.0	PacC ↑
MAA_03460 (XP_007819649)	KFG82099 (ChiMaA5)	95.8	MAPK ↓, Skn7 ↓, PacC ↑
MAA_08154 (XP_007824343)	KFG82955 (ChiMaA6)	93.0	Skn7 ↓, PacC ↑
MAA_10256 (XP_007826445)	KFG81193 (ChiMaA9)	97.1	PacC ↑
Subgroup B chitinases			
MAA_09018 (XP_007825207)	KFG78638 (ChiMaB1)	98.3	PacC ↑
MAA_01212 (XP_007817401)	KFG84275 (ChiMaB2)	82.1	RNS1 ↑
MAA_02744 (XP_007818933)	KFG78862 (ChiMaB3)	94.7	MAPK ↓
MAA_00983 (XP_007817172)	KFG77924 (ChiMaB4)	85.6	PacC ↑
MAA_00415 (XP_007816604)	KFG77460 (ChiMaB5)	74.0	PacC ↑
MAA_09091 (XP_007825280)	KFG80416 (ChiMaB7)	87.7	PacC ↑
Subgroup C chitinases			
MAA_00157 (XP_007816346) ^B^	KFG83700 (ChiMaC1)	48.5	MAPK ↑, COH2 ↑
MAA_10509 (XP_007826698)	KFG84211 (ChiMaC4)	69.3	MAPK ↑
Subgroup D chitinases			
MAA_08315 (XP_007824504)	KFG86059 (ChiMaD1)	92.2	MAPK ↓
GH20 NAGase			
MAA_10080 (XP_007826269)	KFG85702 (MaNAG2)	97.6	PacC↑, RNS1↑

^A^ The effect of the regulator on the expression of target chitinase and GH20 NAGase genes is indicated by an arrow. An (↑) arrow indicates that the expression of the target gene is up-regulated by the regulator, while the (↓) arrow indicates that the expression of the target gene is down-regulated.^B^ The enzyme exhibits sequence similarity to ChiMaC1, ChiMaC2, and ChiMaC3. ChiMaC1 displays the highest sequence similarity (48.5%).

PacC is another regulator of chitinase expression in entomopathogenic fungi. PacC is a pH-dependent transcription factor that governs fungal adaptation to ambient environment. Under acidic pH conditions, PacC exhibits a closed protein conformation that protects the C-terminal domain from protease hydrolysis, and thus PacC remains in the cytoplasm. When pH changes to alkaline, PacC is activated by two proteolysis steps of the C-terminal region, which causes the protein to localize to the nucleus. Inside the nucleus, PacC binds to the consensus sequence 5′-GCCA(A/G)G-3′ (Li et al., 2022a). Deletion of *pacC* in *M. robertsii* resulted in a strain with impaired virulence and reduced adaptation to changes in pH. Additionally, the strain showed reduced chitinase activity; however, protease activity was not affected. *In silico* analysis of the promoter region of *M. robertsii* chitinases revealed the presence of the PacC consensus binding sequence in nine chitinase-encoding genes and one GH20 NAGase (Huang et al., 2015). Homologs of PacC transcription factor and PacC-regulated chitinases and NAGases have been identified in *M. anisopliae* (**Tables 4** and **5**). This suggests similar roles for PacC in *M. anisopliae* (**Figure 4**).

Gao et al. (2011) found that MAP kinase and cAMP dependent protein kinase A (PKA) pathways are up-regulated in *M. anisopliae* after challenging mycelia with cuticle from cockroach and locust, suggesting a role for both kinases in regulating the chitinase gene expression. Nevertheless, the relative contributions of both pathways in regulating chitinase expression were not investigated. Similarly, Chen et al. (2016) have shown that the MAPK cascade regulates the expression of chitinase genes in *M. robertsii* during infection, with two chitinases up-regulated (MAA_00157 and MAA_10509) and four chitinases down-regulated (MAA_08315, MAA_02744, MAA_03460, and MAA_00424). However, the downstream transcription factors that regulate chitinase gene expression were not identified in this study. In addition, the MAPK pathway has been reported to be conserved in several *Metarhizium* species other than *M. anisopliae* (Tang et al., 2022). We identified homologs of MAPK cascade proteins and the MAPK-regulated chitinases in *M. anisopliae* E6 strain (**Tables 4** and **5**).

Several regulators control the expression of chitinase genes in other entomopathogenic fungi. Ron1 is a transcription factor and a key regulator of GlcNAc catabolism (**Figure 4**). In addition, Ron1 plays an important role in fungal virulence, extracellular chitinase activities, and cell wall biosynthesis of the entomopathogenic fungus *B. bassiana* (Qiu et al., 2022). Sre1 is another transcription factor that regulates chitinase gene expression in fungi (Gómez et al., 2020). Deletion of *sre1* in the mycoparasitic fungus *Clonostachys rosea* down-regulated the expression of chitinase genes (Piombo et al., 2024). In addition, RNS1 is a Myb transcription factor that is activated by Fus3 MAPK. Activated RNS1 binds to the 5’-ACCAGAC-3’ sequence in the promoter region and up-regulates the expression of cuticle-degrading genes, including one chitinase and one NAGase (Meng et al., 2021). Ste12 is a transcription factor that is also activated by Fus3 MAPK. Deletion of *ste12* in *M. rileyi* reduced chitin content in conidia; however, the target chitinases were not investigated (Lin et al., 2021). Homologs of Ron1, Sre1, RNS1, and Ste12, and the chitinolytic enzymes that are controlled by these regulators, are conserved in *M. anisopliae* (**Tables 4** and **5**). This suggests a similar role for their homologs in *M. anisopliae* (**Figure 4**); however, the molecular characterization of these proteins is required. Furthermore, two novel transcription factors, designated COH1 and COH2, were shown to regulate chitinases in *M. robertsii*. Both transcription factors are required for fungal colonization of the hemocoel. In the Δ*coh2* mutant, gene expression of three chitinases (MAA_00157, MAA_10456, and MAA_11305) was reported to be down-regulated (Zhang et al., 2021). However, when we ran BLAST analysis of these three chitinases, only a homolog of MAA_00157 was identified in the *M. anisopliae* genome. In contrast, MAA_10456 showed no homology to any known proteins. Our BLAST analysis of MAA_11305 identified a hypothetical protein MANI_008911 (accession number: KFG79813) with no GH18 catalytic domain. Instead, the protein harbors an aminoglycoside 3’-phosphotransferase and Choline kinase domain, which suggests that this protein is not a chitinase. For reviews on the role of transcription factors in entomopathogenic fungi, we recommend the reader to refer to a recent review (Song et al., 2025).

The mechanism that mediates GlcNAc transportation into the cell has been explored in *T. reesei*. Extracellular GlcNAc is imported to the cell via the N‐acetylglucosamine transporter (NGT1), which belongs to the major facilitator superfamily (MFS). Once GlcNAc is internalized in the cell, it activates catabolic gene expression of approximately 2100 genes. Ron1 serves as a major regulator for these genes, while the carbon-catabolite repressor CRE1 is a key repressor (**Figure 4**) (Song et al., 2019; Ullah et al., 2025). The *M. anisopliae* genome harbors a putative homolog of NGT1 (**Table 4**). In addition, the carbon catabolite-repression mechanism has been proposed in *M. anisopliae* based on the fact that chitinase activity is abrogated with increasing concentrations of glucose alone or in combination with chitin (Krieger de Moraes et al., 2003; Barreto et al., 2004). However, these studies lack comprehensive molecular analyses.

## 6 Perspectives and applications of chitinases from *M. anisopliae*

The potential of *M. anisopliae* as a biocontrol agent against agricultural pests have been reviewed by many reports (Ferreira and Soares, 2023; Mesquita et al., 2023). Chitinases from *M. anisopliae* have been extensively characterized at the molecular and biochemical levels; however, relatively few studies have explored their broader applications beyond insect pathogenicity, particularly in plant protection and strain improvement strategies. Emerging research demonstrates that *M. anisopliae* chitinases can be exploited both directly and indirectly to enhance resistance against plant pathogens and insect pests.

Several studies have evaluated the potential of *M. anisopliae* chitinases in engineering transgenic plants with improved resistance to fungal diseases and insect infestations. For example, transient expression of the *chit1* gene from *M. anisopliae* in cotton plants resulted in 9% mortality of adult whiteflies, *Bemisia tabaci*, feeding on the transformed plants (Anwar et al., 2019). Similarly, tobacco plants expressing *chit1* under the control of the CaMV 35S promoter exhibited enhanced resistance to infection by the plant pathogenic fungus *Rhizoctonia solani* (Kern et al., 2010). These findings indicate that *Metarhizium*-derived chitinases can function effectively in heterologous plant systems to confer protection against both fungal and insect threats.

In addition to plant-based approaches, chitinases from *M. anisopliae* have been used to enhance antagonistic activity in mycoparasitic fungi, thereby improving biological control potential. Overexpression of the *chit1* gene in *T. harzianum* significantly increased antagonism against the plant pathogen *Botrytis cinerea* (Li et al., 2012). Likewise, expression of *M. anisopliae* chitinases in *T. koningi* enhanced lethality against the Asian corn borer larvae (Xia et al., 2018). These studies highlight the versatility of *M. anisopliae* chitinases as functional tools for improving fungal biocontrol agents across different host systems.

Beyond these applied perspectives, chitinases play a central mechanistic role in fungal biocontrol efficacy. They are considered key virulence determinants that directly influence the performance of entomopathogenic fungi by mediating degradation of the insect cuticle during host penetration. In *M. anisopliae*, chitinases act synergistically with proteases and lipases to enable successful entry into the insect hemocoel, a prerequisite for infection and host colonization (Ortiz-Urquiza and Keyhani, 2013; Wang and Wang, 2017). The secretion, regulation, and enzymatic activity of chitinases are therefore critical factors governing biocontrol success.

Experimental evidence further demonstrates that enhancing chitinase expression can improve fungal virulence and infection efficiency. Overexpression of the chitinase gene *Bbchit1* in *Beauveria bassiana* resulted in accelerated host mortality and increased pathogenicity (Fang et al., 2005). These results provide proof-of-concept that strengthening cuticle-degrading capacity can enhance the effectiveness of fungal biocontrol agents, although such approaches must be carefully evaluated for ecological safety and regulatory compliance.

Finally, successful biocontrol applications depend not only on the genetic potential but also on the effective enzyme activity at the host surface. Sustained contact between fungal propagules and the insect cuticle is essential for sufficient enzymatic degradation to occur, emphasizing the importance of formulation and application strategies that support chitinase-mediated penetration (Lovett and St. Leger, 2017). Consequently, integrating molecular insights into chitinase function with practical deployment strategies provides a rational framework for improving the consistency, reliability, and performance of *Metarhizium*-based biological control systems (Wang and Wang, 2017).

## 7 Discussion

*M. anisopliae* is a promising entomopathogenic fungus that is commercially used in the biological control of insect pests. Overall, this review emphasizes the diversity of *M. anisopliae* chitinases, and highlights their fungal subgroup distribution, domain architecture, and the conserved mechanisms underlying gene expression. *M. anisopliae* possesses a complex arsenal of chitin-degrading enzymes. Nevertheless, only three chitinases were characterized. Therefore, a significant amount of work is still required to characterize the remaining chitinases. Similarly, the identification of signaling pathways that regulate the expression of chitinase genes remains elusive. Chitinase genes encode enzymes with overlapping activities, and therefore other chitinases can mask the phenotype of the deleted gene, a mechanism known as functional redundancy. Because of the redundancy of chitinases in the genome, it is challenging to identify the precise function of an individual chitinase gene using a single knockout mutant. Therefore, double- or triple-knockout mutants should be generated. However, the disruption of multiple chitinase genes in a single strain is limited by the number of selectable markers currently available. One way to overcome this challenge is by recycling the selectable marker genes using the Cre–loxP recombination system. Another approach is to employ CRISPR–Cas9 technology to simultaneously knock out multiple chitinase genes or to develop a marker-free gene-deletion strategy.

The identification of chitinases and their regulatory proteins can also be achieved via a combination of genomics, transcriptomics, and proteomics approaches, which allow for high-throughput analyses of chitinases and their upstream regulators. For example, investigating the expression profiling of chitinase genes under different insect cuticular components, host developmental stages, or environmental stresses provides insight into the conditions under which chitinase genes are turned on. Virulence assays across multiple insect hosts and environmental conditions reveal context-dependent redundancy. In addition, heterologous expression of *M. anisopliae* chitinases in bacteria, filamentous fungi, and yeast allows for deciphering the biochemical properties of chitinases and their biological function. Transgenic plants that are resistant to insect pests or fungal pathogens can be generated by expressing *M. anisopliae* chitinases in plants. Domain engineering is another approach to analyze the function of domains and engineer chimeric chitinases for industrial degradation of chitin.

## References

[B1] Abdulhameed A. S. Al Omari R. H. Abdullah S. Al-Masud A. A. Abualhaija M. Algburi S. (2025). Nanoarchitectonics of biohybrid polymer nanocomposite of carboxylated chitosan-phthalate/SnO2 nanoparticles for dye wastewater treatment: characterisation, isotherm, kinetic, and adsorption optimisation. Int. J. Environ. Anal. Chem. 105, 6799–6820. doi: 10.1080/03067319.2024.2429792

[B2] Al-Nasrawi H. Shalan N. Abualsoud B. M. Nsairat H. (2024). Preparation, characterization and *in vitro* evaluation of 5-fluorouracil loaded into chitosan–acacia gum nanoparticles. Ther. Deliv. 15, 339–353. doi: 10.4155/tde-2023-0136, PMID: 38469691 PMC11160445

[B3] Andersen S. O. (2009). “ Chapter 94 - exoskeleton,” in Encyclopedia of insects, 2nd ed.Eds. Resh V. H. Cardé R. T. ( Academic Press, San Diego), 339–342. doi: 10.1016/B978-0-12-374144-8.00103-X

[B4] Anwar W. Javed M. A. Shahid A. A. Nawaz K. Akhter A. Ur Rehman M. Z. . (2019). Chitinase genes from Metarhizium anisopliae for the control of whitefly in cotton. R. Soc Open Sci. 6, 190412. doi: 10.1098/rsos.190412, PMID: 31598241 PMC6731705

[B5] Bacik J.-P. Whitworth G. E. Stubbs K. A. Vocadlo D. J. Mark B. L. (2012). Active site plasticity within the glycoside hydrolase nagZ underlies a dynamic mechanism of substrate distortion. Chem. Biol. 19, 1471–1482. doi: 10.1016/j.chembiol.2012.09.016, PMID: 23177201

[B6] Baratto C. M. Dutra V. Boldo J. T. Leiria L. B. Vainstein M. H. Schrank A. (2006). Isolation, Characterization, and Transcriptional Analysis of the Chitinase chi2 Gene (DQ011663) from the Biocontrol Fungus Metarhizium anisopliae var. anisopliae. Curr. Microbiol. 53, 217–221. doi: 10.1007/s00284-006-0078-6, PMID: 16874542

[B7] Baratto C. M. Vanusa da Silva M. Santi L. Passaglia L. Schrank I. S. Vainstein M. H. . (2003). Expression and characterization of the 42 kDa chitinase of the biocontrol fungus Metarhizium anisopliae in Escherichia coli. Can. J. Microbiol. 49, 723–726. doi: 10.1139/w03-085, PMID: 14735222

[B8] Barreto C. C. Staats C. C. Schrank A. Vainstein M. H. (2004). Distribution of chitinases in the entomopathogen metarhizium anisopliae and effect of N-acetylglucosamine in protein secretion. Curr. Microbiol. 48, 102–107. doi: 10.1007/s00284-003-4063-z, PMID: 15057476

[B9] Beys-da-Silva W. O. Santi L. Berger M. Calzolari D. Passos D. O. Guimarães J. A. . (2014). Secretome of the Biocontrol Agent Metarhizium anisopliae Induced by the Cuticle of the Cotton Pest Dysdercus Peruvianus Reveals New Insights into Infection. J. Proteome Res. 13, 2282–2296. doi: 10.1021/pr401204y, PMID: 24702058 PMC4012838

[B10] Bhanu Prakash G. V. S. Padmaja V. Jami S. K. Kirti P. B. (2012). Expression of chitinase genes of Metarhizium anisopliae isolates in lepidopteran pests and on synthetic media. J. Basic Microbiol. 52, 628–635. doi: 10.1002/jobm.201100274, PMID: 22581664

[B11] Bidochka M. J. Kasperski J. E. Wild G. A. (1998). Occurrence of the entomopathogenic fungi Metarhizium anisopliae and Beauveria bassiana in soils from temperate and near-northern habitats. Can. J. Bot. 76, 1198–1204. doi: 10.1139/b98-115

[B12] Bischoff J. F. Rehner S. A. Humber R. A. (2009). A multilocus phylogeny of the Metarhizium anisopliae lineage. Mycologia 101, 512–530. doi: 10.3852/07-202, PMID: 19623931

[B13] Blackwell M. (2011). The Fungi: 1, 2, 3 … 5.1 million species? Am. J. Bot. 98, 426–438. doi: 10.3732/ajb.1000298, PMID: 21613136

[B14] Bogo M. R. Rota C. A. Pinto H. Jr. Ocampos M. Correa C. T. Vainstein M. H. . (1998). A Chitinase Encoding Gene (chit1 Gene) from the Entomopathogen Metarhizium anisopliae: Isolation and Characterization of Genomic and Full-Length cDNA. Curr. Microbiol. 37, 221–225. doi: 10.1007/s002849900368, PMID: 9732526

[B15] Boldo J. T. Amaral K. B. do, Junges A. Pinto P. M. Staats C. C. Vainstein M. H. . (2010). Evidence of alternative splicing of the *chi2* chitinase gene from *Metarhizium anisopliae*. Gene 462, 1–7. doi: 10.1016/j.gene.2010.04.005, PMID: 20406672

[B16] Boldo J. T. Junges A. do Amaral K. B. Staats C. C. Vainstein M. H. Schrank A. (2009). Endochitinase CHI2 of the biocontrol fungus Metarhizium anisopliae affects its virulence toward the cotton stainer bug Dysdercus Peruvianus. Curr. Genet. 55, 551–560. doi: 10.1007/s00294-009-0267-5, PMID: 19649636

[B17] Busk P. K. Lange L. (2015). Classification of fungal and bacterial lytic polysaccharide monooxygenases. BMC Genomics 16, 368. doi: 10.1186/s12864-015-1601-6, PMID: 25956378 PMC4424831

[B18] Butt T. M. Coates C. J. Dubovskiy I. M. Ratcliffe N. A. (2016). “ Chapter nine - entomopathogenic fungi: new insights into host–pathogen interaction,” in Advances in genetics. Eds. Lovett B. St. Leger R. J. (United States: Academic Press). doi: 10.1016/bs.adgen.2016.01.006, PMID: 27131329

[B19] Camargo M. da S. Edwards S. Henrique M. O. Solanki U. Shin T. Y. . (2025). Mycoviruses in entomopathogenic fungi. Viruses 17, 1593. doi: 10.3390/v17121593, PMID: 41472263 PMC12737583

[B20] Catlett N. L. Yoder O. C. Turgeon B. G. (2003). Whole-genome analysis of two-component signal transduction genes in fungal pathogens. Eukaryot. Cell 2, 1151–1161. doi: 10.1128/ec.2.6.1151-1161.2003, PMID: 14665450 PMC326637

[B21] Chen W. Jiang X. Yang Q. (2020). Glycoside hydrolase family 18 chitinases: The known and the unknown. Biotechnol. Adv. 43, 107553. doi: 10.1016/j.bioteChadv.2020.107553, PMID: 32439576

[B22] Chen P.-Y. Lin A. Y.-M. McKittrick J. Meyers M. A. (2008). Structure and mechanical properties of crab exoskeletons. Acta Biomater. 4, 587–596. doi: 10.1016/j.actbio.2007.12.010, PMID: 18299257

[B23] Chen X. Xu C. Qian Y. Liu R. Zhang Q. Zeng G. . (2016). MAPK cascade-mediated regulation of pathogenicity, conidiation and tolerance to abiotic stresses in the entomopathogenic fungus Metarhizium robertsii. Environ. Microbiol. 18, 1048–1062. doi: 10.1111/1462-2920.13198, PMID: 26714892

[B24] Chethana K. W. T. Jayawardena R. S. Chen Y.-J. Konta S. Tibpromma S. Abeywickrama P. D. . (2021). Diversity and function of appressoria. Pathog. Basel Switz. 10, 746. doi: 10.3390/pathogens10060746, PMID: 34204815 PMC8231555

[B25] da Silva M. V. Santi L. Staats C. C. da Costa A. M. Colodel E. M. Driemeier D. . (2005). Cuticle-induced endo/exoacting chitinase CHIT30 from *Metarhizium anisopliae* is encoded by an ortholog of the *chi3* gene. Res. Microbiol. 156, 382–392. doi: 10.1016/j.resmic.2004.10.013, PMID: 15808943

[B26] de la Paz Giménez-Pecci M. Bogo M. R. Santi L. Moraes C. K. Corrêa C. T. Henning Vainstein M. . (2002). Characterization of mycoviruses and analyses of chitinase secretion in the biocontrol fungus metarhizium anisopliae. Curr. Microbiol. 45, 334–339. doi: 10.1007/s00284-002-3678-9, PMID: 12232663

[B27] de Miranda R. P. R. Soares T. K. dos A. Castro D. P. Genta F. A. (2024). General aspects, host interaction, and application of Metarhizium sp. in arthropod pest and vector control. Front. Fungal Biol. 5. doi: 10.3389/ffunb.2024.1456964, PMID: 39634290 PMC11614621

[B28] Ding Y. Wang J. Wang J. Stierhof Y.-D. Robinson D. G. Jiang L. (2012). Unconventional protein secretion. Trends Plant Sci. 17, 606–615. doi: 10.1016/j.tplants.2012.06.004, PMID: 22784825

[B29] Doucet D. Retnakaran A. (2016). “ Targeting cuticular components for pest management,” in *Extracellular composite matrices in arthropods*. Eds. Cohen E. Moussian B. ( Springer International Publishing, Cham), 369–407. doi: 10.1007/978-3-319-40740-1_10

[B30] Fang W. Leng B. Xiao Y. Jin K. Ma J. Fan Y. . (2005). Cloning of beauveria bassiana chitinase gene bbchit1 and its application to improve fungal strain virulence. Appl. Environ. Microbiol. 71 (1), 363–370. doi: 10.1128/AEM.71.1.363-370.2005, PMID: 15640210 PMC544255

[B31] Fassler J. S. West A. H. (2011). Fungal skn7 stress responses and their relationship to virulence. Eukaryot. Cell 10, 156–167. doi: 10.1128/ec.00245-10, PMID: 21131436 PMC3067409

[B32] Ferreira J. M. Soares F. E. (2023). Entomopathogenic fungi hydrolytic enzymes: A new approach to biocontrol? J. Nat. Pestic. Res. 3, 100020. doi: 10.1016/j.napere.2023.100020

[B33] Gao Q. Jin K. Ying S.-H. Zhang Y. Xiao G. Shang Y. . (2011). Genome Sequencing and Comparative Transcriptomics of the Model Entomopathogenic Fungi Metarhizium anisopliae and M. acridum. PloS Genet. 7, e1001264. doi: 10.1371/journal.pgen.1001264, PMID: 21253567 PMC3017113

[B34] Gebremariam A. Chekol Y. Assefa F. (2022). Extracellular enzyme activity of entomopathogenic fungi, Beauveria bassiana and Metarhizium anisopliae and their pathogenicity potential as a bio-control agent against whitefly pests, Bemisia tabaci and Trialeurodes vaporariorum (Hemiptera: Aleyrodidae). BMC Res. Notes 15, 117. doi: 10.1186/s13104-022-06004-4, PMID: 35346347 PMC8961919

[B35] Gómez M. Campusano S. Gutiérrez M. S. Sepúlveda D. Barahona S. Baeza M. . (2020). Sterol regulatory element-binding protein Sre1 regulates carotenogenesis in the red yeast Xanthophyllomyces dendrorhous. J. Lipid Res. 61, 1658–1674. doi: 10.1194/jlr.RA120000975, PMID: 32933952 PMC7707178

[B36] González M. Brito N. González C. (2012). High abundance of Serine/Threonine-rich regions predicted to be hyper-O-glycosylated in the secretory proteins coded by eight fungal genomes. BMC Microbiol. 12, 213. doi: 10.1186/1471-2180-12-213, PMID: 22994653 PMC3579731

[B37] Gorb S. (2001). “ Insect cuticle: structure and properties of the material,” in Attachment devices of insect cuticle ( Springer Netherlands, Dordrecht), 9–19. doi: 10.1007/0-306-47515-4_2

[B38] Gortari M. C. Hours R. A. (2008). Fungal chitinases and their biological role in the antagonism onto nematode eggs. A review. Mycol. Prog. 7, 221–238. doi: 10.1007/s11557-008-0571-3

[B39] Gruber S. Kubicek C. P. Seidl-Seiboth V. (2011). Differential regulation of orthologous chitinase genes in mycoparasitic *Trichoderma* species. Appl. Environ. Microbiol. 77, 7217–7226. doi: 10.1128/AEM.06027-11, PMID: 21856825 PMC3194852

[B40] Gruber S. Seidl-Seiboth V. (2012). Self versus non-self: fungal cell wall degradation in *Trichoderma*. Microbiology 158, 26–34. doi: 10.1099/mic.0.052613-0, PMID: 21873410

[B41] Guo J. Zhang P. Wu N. Liu W. Liu Y. Jin H. . (2024). Transfection of entomopathogenic Metarhizium species with a mycovirus confers hypervirulence against two lepidopteran pests. Proc. Natl. Acad. Sci. 121, e2320572121. doi: 10.1073/pnas.2320572121, PMID: 38885380 PMC11214047

[B42] Häkkinen M. Arvas M. Oja M. Aro N. Penttilä M. Saloheimo M. . (2012). Re-annotation of the CAZy genes of *Trichoderma reesei* and transcription in the presence of lignocellulosic substrates. Microb. Cell Factories 11, 134. doi: 10.1186/1475-2859-11-134, PMID: 23035824 PMC3526510

[B43] Hamid R. Khan M. A. Ahmad M. Ahmad M. M. Abdin M. Z. Musarrat J. . (2013). Chitinases: an update. J. Pharm. Bioallied Sci. 5, 21–29. doi: 10.4103/0975-7406.106559, PMID: 23559820 PMC3612335

[B44] Han B. Zhou K. Li Z. Sun B. Ni Q. Meng X. . (2016). Characterization of the first fungal glycosyl hydrolase family 19 chitinase (NbchiA) from nosema bombycis (Nb). J. Eukaryot. Microbiol. 63, 37–45. doi: 10.1111/jeu.12246, PMID: 26108336

[B45] Hartl L. Zach S. Seidl-Seiboth V. (2012). Fungal chitinases: diversity, mechanistic properties and biotechnological potential. Appl. Microbiol. Biotechnol. 93, 533–543. doi: 10.1007/s00253-011-3723-3, PMID: 22134638 PMC3257436

[B46] Henrissat B. Davies G. (1997). Structural and sequence-based classification of glycoside hydrolases. Curr. Opin. Struct. Biol. 7, 637–644. doi: 10.1016/S0959-440X(97)80072-3, PMID: 9345621

[B47] Hough B. Steenkamp E. Wingfield B. Read D. (2023). Fungal viruses unveiled: A comprehensive review of mycoviruses. Viruses 15, 1202. doi: 10.3390/v15051202, PMID: 37243288 PMC10224137

[B48] Huang W. Shang Y. Chen P. Gao Q. Wang C. (2015). MrpacC regulates sporulation, insect cuticle penetration and immune evasion in Metarhizium robertsii. Environ. Microbiol. 17, 994–1008. doi: 10.1111/1462-2920.12451, PMID: 24612440

[B49] Huang Q.-S. Xie X.-L. Liang G. Gong F. Wang Y. Wei X.-Q. . (2012). The GH18 family of chitinases: their domain architectures, functions and evolutions. Glycobiology 22, 23–34. doi: 10.1093/glycob/cwr092, PMID: 21750098

[B50] Intra J. Pavesi G. Horner D. S. (2008). Phylogenetic analyses suggest multiple changes of substrate specificity within the Glycosyl hydrolase 20 family. BMC Evol. Biol. 8, 214. doi: 10.1186/1471-2148-8-214, PMID: 18647384 PMC2492878

[B51] Iwanicki N. S. Botelho A. B. R. Z. Klingen I. Júnior I. D. Rossmann S. Lysøe E. (2022). Genomic signatures and insights into host niche adaptation of the entomopathogenic fungus Metarhizium humberi. G3 GenesGenomesGenetics 12, jkab416. doi: 10.1093/g3journal/jkab416, PMID: 34865006 PMC9210286

[B52] Izadi H. Asadi H. Bemani M. (2025). Chitin: a comparison between its main sources. Front. Mater. 12. doi: 10.3389/fmats.2025.1537067

[B53] Jiang C. Li L. Li J. (2025). Characterization of a β-N-acetylhexosaminidase CgNagase20 from Chaetomium globosum with antifungal activity against Fusarium sporotrichioides. World J. Microbiol. Biotechnol. 41, 308. doi: 10.1007/s11274-025-04524-2, PMID: 40788452

[B54] Jones M. Kujundzic M. John S. Bismarck A. (2020). Crab vs. Mushroom: A review of crustacean and fungal chitin in wound treatment. Mar. Drugs 18, 64. doi: 10.3390/md18010064, PMID: 31963764 PMC7024172

[B55] Junges Â. Boldo J. T. Souza B. K. Guedes R. L. M. Sbaraini N. Kmetzsch L. . (2014). Genomic analyses and transcriptional profiles of the glycoside hydrolase family 18 genes of the entomopathogenic fungus metarhizium anisopliae. PloS One 9, e107864. doi: 10.1371/journal.pone.0107864, PMID: 25232743 PMC4169460

[B56] Kappel L. Gaderer R. Flipphi M. Seidl-Seiboth V. (2016). The N-acetylglucosamine catabolic gene cluster in *Trichoderma reesei* is controlled by the Ndt80-like transcription factor RON1. Mol. Microbiol. 99, 640–657. doi: 10.1111/mmi.13256, PMID: 26481444 PMC4950302

[B57] Kern M. F. Maraschin S. de F. Vom Endt D. Schrank A. Vainstein M. H. . (2010). Expression of a Chitinase Gene from Metarhizium anisopliae in Tobacco Plants Confers Resistance against Rhizoctonia solani. Appl. Biochem. Biotechnol. 160, 1933–1946. doi: 10.1007/s12010-009-8701-1, PMID: 19593671

[B58] Khachatourians G. G. Qazi S. S. (2008). “ Entomopathogenic fungi: biochemistry and molecular biology,” in Human and animal relationships. Eds. Brakhage A. A. Zipfel P. F. ( Springer, Berlin, Heidelberg), 33–61. doi: 10.1007/978-3-540-79307-6_3

[B59] Kobmoo N. Mongkolsamrit S. Khonsanit A. Cedeño-Sanchez M. Arnamnart N. Noisripoom W. . (2024). Integrative taxonomy of Metarhizium anisopliae species complex, based on phylogenomics combined with morphometrics, metabolomics, and virulence data. IMA Fungus 15, 30. doi: 10.1186/s43008-024-00154-9, PMID: 39261927 PMC11389511

[B60] Krieger de Moraes C. Schrank A. Vainstein M. H. (2003). Regulation of Extracellular Chitinases and proteases in the entomopathogen and acaricide metarhizium anisopliae. Curr. Microbiol. 46, 0205–0210. doi: 10.1007/s00284-002-3863-x, PMID: 12567244

[B61] Kubicek C. P. Herrera-Estrella A. Seidl-Seiboth V. Martinez D. A. Druzhinina I. S. Thon M. . (2011). Comparative genome sequence analysis underscores mycoparasitism as the ancestral life style of *Trichoderma*. Genome Biol. 12, R40. doi: 10.1186/gb-2011-12-4-r40, PMID: 21501500 PMC3218866

[B62] Kumar K. K. Sridhar J. Murali-Baskaran R. K. Senthil-Nathan S. Kaushal P. Dara S. K. . (2019). Microbial biopesticides for insect pest management in India: Current status and future prospects. J. Invertebr. Pathol. 165, 74–81. doi: 10.1016/j.jip.2018.10.008, PMID: 30347206

[B63] Kumirska J. Weinhold M. X. Thöming J. Stepnowski P. (2011). Biomedical activity of chitin/chitosan based materials—Influence of physicochemical properties apart from molecular weight and degree of N-acetylation. Polymers 3, 1875–1901. doi: 10.3390/polym3041875

[B64] Langner T. Göhre V. (2016). Fungal chitinases: function, regulation, and potential roles in plant/pathogen interactions. Curr. Genet. 62, 243–254. doi: 10.1007/s00294-015-0530-x, PMID: 26527115

[B65] Li B. Chen Y. Tian S. (2022a). Function of pH-dependent transcription factor PacC in regulating development, pathogenicity, and mycotoxin biosynthesis of phytopathogenic fungi. FEBS J. 289, 1723–1730. doi: 10.1111/febs.15808, PMID: 33751796

[B66] Li Y. Y. Tang J. Fu K. H. Gao S. G. Wu Q. Chen J. (2012). Construction of transgenic *Trichoderma koningi* with chit42 of Metarhizium anisopliae and analysis of its activity against the Asian corn borer. J. Environ. Sci. Health Part B 47, 622–630. doi: 10.1080/03601234.2012.668455, PMID: 22560024

[B67] Li J. Xia Y. (2022). Host–pathogen interactions between metarhizium spp. and locusts. J. Fungi 8, 602. doi: 10.3390/jof8060602, PMID: 35736085 PMC9224550

[B68] Li C.-C. Yi H. Wang Y.-M. Tang X.-Y. Zhu Y.-B. Song Y.-J. . (2022b). Nucleotide binding as an allosteric regulatory mechanism for Akkermansia muciniphila β-N-acetylhexosaminidase Am2136. Gut Microbes 14, 2143221. doi: 10.1080/19490976.2022.2143221, PMID: 36394293 PMC9673926

[B69] Lin Y. Wang J. Yang K. Fan L. Wang Z. Yin Y. (2021). Regulation of conidiation, polarity growth, and pathogenicity by MrSte12 transcription factor in entomopathogenic fungus, *Metarhizium rileyi*. Fungal Genet. Biol. 155, 103612. doi: 10.1016/j.fgb.2021.103612, PMID: 34303798

[B70] Liu T. Yan J. Yang Q. (2012). Comparative biochemistry of GH3, GH20 and GH84 β-N-acetyl-Dhexosaminidases and recent progress in selective inhibitor discovery. Curr. Drug Targets 13, 512–525. doi: 10.2174/138945012799499730, PMID: 22280348

[B71] López-Mondéjar R. Catalano V. Kubicek C. P. Seidl V. (2009). The β-N-acetylglucosaminidases NAG1 and NAG2 are essential for growth of *Trichoderma atroviride* on chitin. FEBS J. 276, 5137–5148. doi: 10.1111/j.1742-4658.2009.07211.x, PMID: 19674110

[B72] Lovett B. St. Leger R. J. (2017). The insect pathogens. Microbiol. Spectr. 5, 1128. doi: 10.1128/microbiolspec.funk-0001-2016, PMID: 28256192 PMC11687457

[B73] Ma M. Luo J. Li C. Eleftherianos I. Zhang W. Xu L. (2024). A life-and-death struggle: interaction of insects with entomopathogenic fungi across various infection stages. Front. Immunol. 14. doi: 10.3389/fimmu.2023.1329843, PMID: 38259477 PMC10800808

[B74] Macdonald S. S. Blaukopf M. Withers S. G. (2015). *N*-acetylglucosaminidases from CAZy family GH3 are really glycoside phosphorylases, thereby explaining their use of histidine as an acid/base catalyst in place of glutamic acid*. J. Biol. Chem. 290, 4887–4895. doi: 10.1074/jbc.M114.621110, PMID: 25533455 PMC4335228

[B75] Mei Z. Kuzhir P. Godeau G. (2024). Update on chitin and chitosan from insects: sources, production, characterization, and biomedical applications. Biomimetics 9, 297. doi: 10.3390/biomimetics9050297, PMID: 38786507 PMC11118814

[B76] Meng Y. Zhang X. Tang D. Chen X. Zhang D. Chen J. . (2021). A novel nitrogen and carbon metabolism regulatory cascade is implicated in entomopathogenicity of the fungus metarhizium robertsii. mSystems 6, 10.1128/msystems.00499–21. doi: 10.1128/msystems.00499-21, PMID: 34156296 PMC8269237

[B77] Mesquita E. Hu S. Lima T. B. Golo P. S. Bidochka M. J. (2023). Utilization of Metarhizium as an insect biocontrol agent and a plant bioinoculant with special reference to Brazil. Front. Fungal Biol. 4. doi: 10.3389/ffunb.2023.1276287, PMID: 38186633 PMC10768067

[B78] Moazami N. (2011). “ Biological control,” in Comprehensive biotechnology, 2nd ed.Ed. Moo-Young M. ( Academic Press, Burlington), 731–739. doi: 10.1016/B978-0-08-088504-9.00235-X

[B79] Moonjely S. Bidochka M. J. (2019). Generalist and specialist *Metarhizium* insect pathogens retain ancestral ability to colonize plant roots. Fungal Ecol. 41, 209–217. doi: 10.1016/j.funeco.2019.06.004

[B80] Mukherjee P. K. Horwitz B. A. Herrera-Estrella A. Schmoll M. Kenerley C. M. (2013). *Trichoderma* research in the genome era. Annu. Rev. Phytopathol. 51, 105–129. doi: 10.1146/annurev-phyto-082712-102353, PMID: 23915132

[B81] Muschiol J. Vuillemin M. Meyer A. S. Zeuner B. (2020). β-N-acetylhexosaminidases for carbohydrate synthesis via trans-glycosylation. Catalysts 10, 365. doi: 10.3390/catal10040365

[B82] Nishi O. (2024). Phylogenetic classification and physiological and ecological traits of Metarhizium spp. Mycoscience 65, 235–243. doi: 10.47371/mycosci.2024.07.001, PMID: 39831172 PMC11739784

[B83] Oliveira E. S. Junges Â. Sbaraini N. Andreis F. C. Thompson C. E. Staats C. C. . (2018). Molecular evolution and transcriptional profile of GH3 and GH20 β-N-acetylglucosaminidases in the entomopathogenic fungus Metarhizium anisopliae. Genet. Mol. Biol. 41, 843–857. doi: 10.1590/1678-4685-GMB-2017-0363, PMID: 30534852 PMC6415606

[B84] Ortiz-Urquiza A. Keyhani N. O. (2013). Action on the surface: entomopathogenic fungi versus the insect cuticle. Insects 4, 357–374. doi: 10.3390/insects4030357, PMID: 26462424 PMC4553469

[B85] Ortiz-Urquiza A. Keyhani N. O. (2016). Molecular genetics of beauveria bassiana infection of insects. Adv. Genet. 94, 165–249. doi: 10.1016/bs.adgen.2015.11.003, PMID: 27131326

[B86] Oyeleye A. Normi Y. M. (2018). Chitinase: diversity, limitations, and trends in engineering for suitab le applications. Biosci. Rep. 38, BSR2018032300. doi: 10.1042/BSR20180323, PMID: 30042170 PMC6131217

[B87] Palma-Guerrero J. Jansson H.-B. Salinas J. Lopez-Llorca L. V. (2008). Effect of chitosan on hyphal growth and spore germination of plant pathogenic and biocontrol fungi. J. Appl. Microbiol. 104, 541–553. doi: 10.1111/j.1365-2672.2007.03567.x, PMID: 17927761

[B88] Pattemore J. A. Hane J. K. Williams A. H. Wilson B. A. Stodart B. J. Ash G. J. (2014). The genome sequence of the biocontrol fungus Metarhizium anisopliae and comparative genomics of Metarhizium species. BMC Genomics 15, 660. doi: 10.1186/1471-2164-15-660, PMID: 25102932 PMC4133081

[B89] Peng Y. Wang L. Gao Y. Ye L. Xu H. Li S. . (2020). Identification and characterization of the glycoside hydrolase family 18 genes from the entomopathogenic fungus Isaria cicadae genome. Can. J. Microbiol. 66, 274–287. doi: 10.1139/cjm-2019-0129, PMID: 31961710

[B90] Perinotto W. M. S. Golo P. S. Coutinho Rodrigues C. J. B. Sá F. A. Santi L. Beys da Silva W. O. . (2014). Enzymatic activities and effects of mycovirus infection on the virulence of *Metarhizium anisopliae* in *Rhipicephalus microplus*. Vet. Parasitol. 203, 189–196. doi: 10.1016/j.vetpar.2014.02.011, PMID: 24661809

[B91] Pinto A. de S. Barreto C. C. Vainstein M. H. Schrank A. Ulhoa C. J. (1997). Purification and characterization of an extracellular chitinase from the entomopathogen Metarhizium anisopliae. Can. J. Microbiol. 43, 322–327. doi: 10.1139/m97-045

[B92] Piombo E. Tzelepis G. Ruus A. G. Rafiei V. Jensen D. F. Karlsson M. . (2024). Sterol regulatory element-binding proteins mediate intrinsic fungicide tolerance and antagonism in the fungal biocontrol agent *Clonostachys rosea* IK726. Microbiol. Res. 289, 127922. doi: 10.1016/j.micres.2024.127922, PMID: 39368255

[B93] Poria V. Rana A. Kumari A. Grewal J. Pranaw K. Singh S. (2021). Current perspectives on chitinolytic enzymes and their agro-industrial applications. Biology 10, 1319. doi: 10.3390/biology10121319, PMID: 34943233 PMC8698876

[B94] Qiu L. Song J.-Z. Li J. Zhang T.-S. Li Z. Hu S.-J. . (2022). The transcription factor Ron1 is required for chitin metabolism, asexual development and pathogenicity in *Beauveria bassiana*, an entomopathogenic fungus. Int. J. Biol. Macromol. 206, 875–885. doi: 10.1016/j.ijbiomac.2022.03.037, PMID: 35278517

[B95] Rajput M. Kumar M. Pareek N. (2022). Myco-chitinases as versatile biocatalysts for translation of coastal residual resources to eco-competent chito-bioactives. Fungal Biol. Rev. 41, 52–69. doi: 10.1016/j.fbr.2022.04.001

[B96] Rustiguel C. B. Rosa J. C. Jorge J. A. de Oliveira A. H. C. Guimarães L. H. S. (2016). Secretome Analysis of Metarhizium anisopliae Under Submerged Conditions Using Bombyx mori Chrysalis to Induce Expression of Virulence-Related Proteins. Curr. Microbiol. 72, 220–227. doi: 10.1007/s00284-015-0943-2, PMID: 26597214

[B97] Santi L. Beys da Silva W. O. Berger M. Guimarães J. A. Schrank A. Vainstein M. H. (2010a). Conidial surface proteins of *Metarhizium anisopliae*: Source of activities related with toxic effects, host penetration and pathogenesis. Toxicon 55, 874–880. doi: 10.1016/j.toxicon.2009.12.012, PMID: 20034509

[B98] Santi L. Silva W. O. B. Pinto A. F. M. Schrank A. Vainstein M. H. (2010b). *Metarhizium anisopliae* host–pathogen interaction: differential immunoproteomics reveals proteins involved in the infection process of arthropods. Fungal Biol. 114, 312–319. doi: 10.1016/j.funbio.2010.01.006, PMID: 20943140

[B99] Santos A. C. da S. Lopes R. da S. de Oliveira L. G. Diniz A. G. . (2022). “ Entomopathogenic fungi: current status and prospects,” in New and future development in biopesticide research: biotechnological exploration. Eds. Mandal S. D. Ramkumar G. Karthi S. Jin F. ( Springer Nature, Singapore), 55–91. doi: 10.1007/978-981-16-3989-0_2

[B100] Sbaraini N. Junges Â. de Oliveira E. S. Webster A. Vainstein M. H. Staats C. C. . (2021). The deletion of chiMaD1, a horizontally acquired chitinase of Metarhizium anisopliae, led to higher virulence towards the cattle tick (Rhipicephalus microplus). FEMS Microbiol. Lett. 368, fnab066. doi: 10.1093/femsle/fnab066, PMID: 34100915

[B101] Schaller G. E. Shiu S.-H. Armitage J. P. (2011). Two-component systems and their co-option for eukaryotic signal transduction. Curr. Biol. 21, R320–R330. doi: 10.1016/j.cub.2011.02.045, PMID: 21549954

[B102] Schrank A. Vainstein M. H. (2010). *Metarhizium anisopliae* enzymes and toxins. Toxicon 56, 1267–1274. doi: 10.1016/j.toxicon.2010.03.008, PMID: 20298710

[B103] Screen S. E. Hu G. St Leger R. J. (2001). Transformants of Metarhizium anisopliae sf. anisopliae overexpressing chitinase from Metarhizium anisopliae sf. acridum show early induction of native chitinase but are not altered in pathogenicity to Manduca sexta. J. Invertebr. Pathol. 78, 260–266. doi: 10.1006/jipa.2001.5067, PMID: 12009808

[B104] Seidl V. (2008). Chitinases of filamentous fungi: a large group of diverse proteins with multiple physiological functions. Fungal Biol. Rev. 22, 36–42. doi: 10.1016/j.fbr.2008.03.002

[B105] Seidl V. Huemer B. Seiboth B. Kubicek C. P. (2005). A complete survey of *Trichoderma chitinases* reveals three distinct subgroups of family 18 chitinases. FEBS J. 272, 5923–5939. doi: 10.1111/j.1742-4658.2005.04994.x, PMID: 16279955

[B106] Seidl-Seiboth V. Ihrmark K. Druzhinina I. Karlsson M. (2014). “ Molecular evolution of *Trichoderma chitinases*,” in Biotechnology and biology of trichoderma. Eds. Gupta V. K. Schmoll M. Herrera-estrella A. Upadhyay R. S. Druzhinina I. Tuohy M. G. , (Amsterdam: Elsevier) 67–78. doi: 10.1016/B978-0-444-59576-8.00005-9

[B107] Shang Y. Chen P. Chen Y. Lu Y. Wang C. (2015). MrSkn7 controls sporulation, cell wall integrity, autolysis, and virulence in metarhizium robertsii. Eukaryot. Cell 14, 396–405. doi: 10.1128/ec.00266-14, PMID: 25710964 PMC4385802

[B108] Song H.-J. Li X.-F. Pei X.-R. Sun Z.-B. Pan H.-X. (2025). Transcription factors in biocontrol fungi. J. Fungi 11, 223. doi: 10.3390/jof11030223, PMID: 40137261 PMC11943155

[B109] Song Z. Shen L. Yin Y. Tan W. Shao C. Xu J. . (2015). Role of two Nomuraea rileyi transmembrane sensors Sho1p and Sln1p in adaptation to stress due to changing culture conditions during microsclerotia development. World J. Microbiol. Biotechnol. 31, 477–485. doi: 10.1007/s11274-015-1801-x, PMID: 25595731

[B110] Song D. Shi Y. Ji H. Xia Y. Peng G. (2019). The maCreA gene regulates normal conidiation and microcycle conidiation in metarhizium acridum. Front. Microbiol. 10. doi: 10.3389/fmicb.2019.01946, PMID: 31497008 PMC6713048

[B111] Song Z. Yin Y. Jiang S. Liu J. Chen H. Wang Z. (2013). Comparative transcriptome analysis of microsclerotia development in Nomuraea rileyi. BMC Genomics 14, 411. doi: 10.1186/1471-2164-14-411, PMID: 23777366 PMC3698084

[B112] Staats C. C. Junges Â. Guedes R. L. M. Thompson C. E. de Morais G. L. Boldo J. T. . (2014). Comparative genome analysis of entomopathogenic fungi reveals a complex set of secreted proteins. BMC Genomics 15, 822. doi: 10.1186/1471-2164-15-822, PMID: 25263348 PMC4246632

[B113] Staats C. C. Kmetzsch L. Lubeck I. Junges A. Vainstein M. H. Schrank A. (2013). *Metarhizium anisopliae* chitinase CHIT30 is involved in heat-shock stress and contributes to virulence against *Dysdercus Peruvianus*. Fungal Biol. 117, 137–144. doi: 10.1016/j.funbio.2012.12.006, PMID: 23452951

[B114] St. Leger R. J. Cooper R. M. Charnley A. K. (1991). Characterization of chitinase and chitobiase produced by the entomopathogenic fungus *Metarhizium anisopliae*. J. Invertebr. Pathol. 58, 415–426. doi: 10.1016/0022-2011(91)90188-V

[B115] St. Leger R. J. Joshi L. Bidochka M. J. Rizzo N. W. Roberts D. W. (1996). Characterization and Ultrastructural Localization of Chitinases from Metarhizium anisopliae, M. flavoviride, and Beauveria bassiana during Fungal Invasion of Host (Manduca sexta) Cuticle. Appl. Environ. Microbiol. 62, 907–912. doi: 10.1128/aem.62.3.907-912.1996, PMID: 16535278 PMC1388803

[B116] St. Leger R. J. Wang J. B. (2020). Metarhizium: jack of all trades, master of many. Open Biol. 10, 200307. doi: 10.1098/rsob.200307, PMID: 33292103 PMC7776561

[B117] Takaya N. Yamazaki D. Horiuchi H. Ohta A. Takagi M. (1998). Cloning and characterization of a chitinase-encoding gene (chiA) from Aspergillus nidulans, disruption of which decreases germination frequency and hyphal growth. Biosci. Biotechnol. Biochem. 62, 60–65. doi: 10.1271/bbb.62.60, PMID: 9501518

[B118] Talamantes D. Biabini N. Dang H. Abdoun K. Berlemont R. (2016). Natural diversity of cellulases, xylanases, and chitinases in bacteria. Biotechnol. Biofuels 9, 133. doi: 10.1186/s13068-016-0538-6, PMID: 27366206 PMC4928363

[B119] Tang D. Tang X. Fang W. (2022). New downstream signaling branches of the mitogen-activated protein kinase cascades identified in the insect pathogenic and plant symbiotic fungus metarhizium robertsii. Front. Fungal Biol. 3. doi: 10.3389/ffunb.2022.911366, PMID: 37746179 PMC10512405

[B120] Tang X. Wang X. Cheng X. Wang X. Fang W. (2025). Metarhizium fungi as plant symbionts. New Plant Prot. 2, e23. doi: 10.1002/npp2.23

[B121] Ubhayasekera W. Karlsson M. (2012). Bacterial and fungal chitinase chiJ orthologs evolve under different selective constraints following horizontal gene transfer. BMC Res. Notes 5, 581. doi: 10.1186/1756-0500-5-581, PMID: 23095575 PMC3506478

[B122] Ullah S. F. Oreb M. Boles E. Srivastava V. Seidl-Seiboth V. Seiboth B. . (2025). N-acetylglucosamine sensing in the filamentous soil fungus *Trichoderma reesei*. FEBS J. 292, 3072–3090. doi: 10.1111/febs.70015, PMID: 39954246 PMC12176254

[B123] Val-Cid C. Biarnés X. Faijes M. Planas A. (2015). Structural-functional analysis reveals a specific domain organization in family GH20 hexosaminidases. PloS One 10, e0128075. doi: 10.1371/journal.pone.0128075, PMID: 26024355 PMC4449183

[B124] Vincent J. F. V. (2001). “ Cuticle,” in Encyclopedia of materials: science and technology. Eds. Buschow K. H. J. Cahn R. W. Flemings M. C. Ilschner B. Kramer E. J. Mahajan S. ( Elsevier, Oxford), 1924–1928. doi: 10.1016/B0-08-043152-6/00350-8

[B125] Viterbo A. Haran S. Friesem D. Ramot O. Chet I. (2001). Antifungal activity of a novel endochitinase gene (chit36) from *Trichoderma harzianum* Rifai TM. FEMS Microbiol. Lett. 200, 169–174. doi: 10.1111/j.1574-6968.2001.tb10710.x, PMID: 11425470

[B126] Vittori M. (2024). Structural diversity of crustacean exoskeletons and its implications for biomimetics. Interface Focus 14, 20230075. doi: 10.1098/rsfs.2023.0075, PMID: 38618234 PMC11008965

[B127] Wang C. Wang S. (2017). Insect pathogenic fungi: genomics, molecular interactions, and genetic improvements. Annu. Rev. Entomol. 62, 73–90. doi: 10.1146/annurev-ento-031616-035509, PMID: 27860524

[B128] Wang P. Yang G. Shi N. Zhao C. Hu F. Coutts R. H. A. . (2023). A novel partitivirus orchestrates conidiation, stress response, pathogenicity, and secondary metabolism of the entomopathogenic fungus Metarhizium majus. PloS Pathog. 19, e1011397. doi: 10.1371/journal.ppat.1011397, PMID: 37216409 PMC10237674

[B129] Xia H. Li Y. Y. Liu Z. C. Li Y. Q. Chen J. (2018). Transgenic Expression of chit42 gene from Metarhizium anisopliae in *Trichoderma harzianum* Enhances Antagonistic Activity against Botrytis cinerea. Mol. Biol. 52, 668–675. doi: 10.1134/S002689331805014X, PMID: 30363052

[B130] Xiao G. Ying S.-H. Zheng P. Wang Z.-L. Zhang S. Xie X.-Q. . (2012). Genomic perspectives on the evolution of fungal entomopathogenicity in Beauveria bassiana. Sci. Rep. 2, 483. doi: 10.1038/srep00483, PMID: 22761991 PMC3387728

[B131] Yamazaki H. Tanaka A. Kaneko J. Ohta A. Horiuchi H. (2008). Aspergillus nidulans ChiA is a glycosylphosphatidylinositol (GPI)-anchored chitinase specifically localized at polarized growth sites. Fungal Genet. Biol. 45, 963–972. doi: 10.1016/j.fgb.2008.02.008, PMID: 18420434

[B132] Yang S. Song S. Yan Q. Fu X. Jiang Z. Yang X. (2014). Biochemical characterization of the first fungal glycoside hydrolyase family 3 β-N-acetylglucosaminidase from rhizomucor miehei. J. Agric. Food Chem. 62, 5181–5190. doi: 10.1021/jf500912b, PMID: 24811866

[B133] Zhang Z. Guo W. Lu Y. Kang Q. Sui L. Liu H. . (2023). Hypovirulence-associated mycovirus epidemics cause pathogenicity degeneration of Beauveria bassiana in the field. Virol. J. 20, 255. doi: 10.1186/s12985-023-02217-6, PMID: 37924080 PMC10623766

[B134] Zhang X. Meng Y. Huang Y. Zhang D. Fang W. (2021). A novel cascade allows Metarhizium robertsii to distinguish cuticle and hemocoel microenvironments during infection of insects. PloS Biol. 19, e3001360. doi: 10.1371/journal.pbio.3001360, PMID: 34347783 PMC8366996

